# Labeling of CC Chemokine Receptor 2 with a Versatile Intracellular Allosteric Probe

**DOI:** 10.1021/acschembio.4c00439

**Published:** 2024-08-26

**Authors:** Lisa S. den Hollander, Bert L.H. Beerkens, Sebastian Dekkers, Jacobus P.D. van Veldhoven, Natalia V. Ortiz Zacarías, Cas van der Horst, Elisabeth G. Sieders, Bert de Valk, Jianhui Wang, Adriaan P. IJzerman, Daan van der Es, Laura H. Heitman

**Affiliations:** Leiden Academic Centre for Drug Research, Division of Medicinal Chemistry, Leiden 2333 CC, The Netherlands

## Abstract

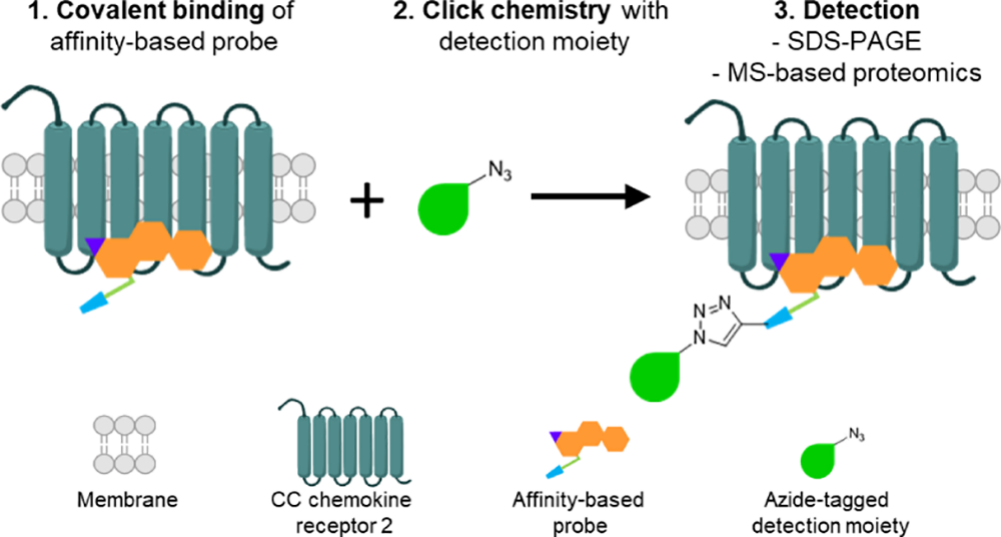

Interest in affinity-based probes (AfBPs) as novel tools to interrogate G protein-coupled receptors (GPCRs) has gained traction in recent years. AfBPs represent an interesting and more versatile alternative to antibodies. In the present study, we report the development and validation of AfBPs that target the intracellular allosteric pocket of CCR2, a GPCR of interest for the development of therapies targeting autoimmune and inflammatory diseases and also cancer. Owing to the two-step labeling process of these CCR2 AfBPs through the incorporation of a click handle, we were successful in applying our most efficient probe in a variety of *in vitro* experiments and making use of multiple different detection techniques, such as SDS-PAGE and LC/MS-based proteomics. Collectively, this novel probe shows high selectivity, versatility, and applicability. Hence, this is a valuable alternative for CCR2-targeting antibodies and other traditional tool compounds and could aid in target validation and engagement in drug discovery.

## Introduction

Activity-based protein profiling (ABPP), as developed by the laboratories of Cravatt and Bogyo, is a method in which specific enzymes are labeled in biological samples using small molecules for target profiling.^1,2^ These small molecules are dubbed activity-based probes (ABPs) and are composed of elements that facilitate the targeting and covalent binding to the active site of an enzyme.^3^ When proteins have no inherent catalytic activity and hence no available active site, as is the case for G protein-coupled receptors (GPCRs), selective targeting relies on a ligand’s high affinity for the target. Covalent interactions with specific amino acids in the ligand binding pocket can still be achieved by incorporating a more chemoreactive or photoreactive warhead in the design of the ligand. A shortage of adequate antibodies has slowed research into GPCR structure, function and biochemical mechanisms.^4^ Affinity-based probes (AfBPs), high-affinity ligands decorated with a reactive group, could potentially fill this gap in GPCR research.^5−14^

**Figure 1:**
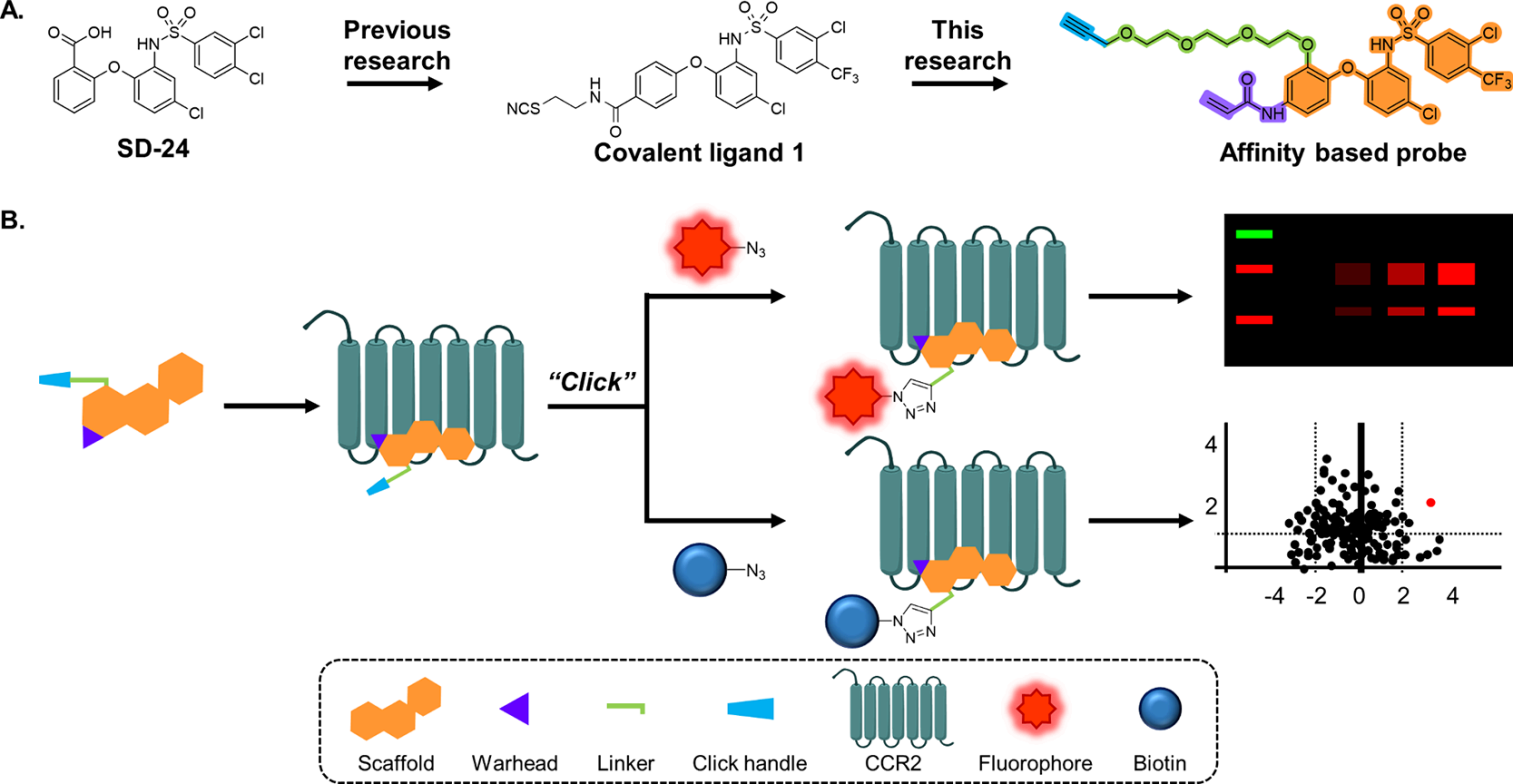
Workflow of this study. (A) Design of affinity-based probes for the intracellular allosteric binding site of CCR2. Ligands are based on the intracellular CCR2-targeting ligand SD-24, which was previously modified into the first covalent ligand for CCR2. Here, the scaffold was decorated with a click handle containing a variety of linker lengths and multiple warheads to yield a number of affinity-based chemical probes. (B) Probes were incubated with cell membranes transiently expressing CCR2 to form a covalent interaction between the warhead and a cysteine in the intracellular allosteric binding pocket. Next, either an azide-tagged fluorophore or azide-tagged biotin was affixed onto the probe’s click handle via CuI-catalyzed click chemistry to form a stable triazole connection. Finally, detection of the probe using in-gel fluorescence for fluorophore-conjugated or affinity-based pull-down proteomics for biotin-conjugated probes was achieved.

Detection of proteins with ABPs and AfBPs is accomplished through the incorporation of a reporter tag (e.g., a fluorophore). This could either be *a priori* incorporation for one-step probes or through incorporation of a ligation handle to which a reporter tag can be attached using bio-orthogonal chemistry for two-step probes.^15,16^ Such reactions between the reporter tag and ligation handle proceed with high selectivity and efficiency and have the possibility to be performed in complex biological environments. Although several strategies for such ligations are available, the AfBPs reported in this study have been designed to be compatible with copper-catalyzed azide–alkyne cycloadditions (CuAAC).^16^ An azide-conjugated reporter moiety selectively reacts with an alkyne “click”-handle via copper-catalyzed [3 + 2] cycloaddition to form a 1,2,3-triazole (Figure 1).

The advantage of a two-step versus one-step probe in our setup is twofold: (1) Structural modification of the intended probe is minimized compared to the incorporation of a bulky reporter tag, and thereby, the loss of affinity or selectivity that these tags can induce is minimized,^17^ and (2) a two-step reaction provides a means to use a single probe to enable a wide range of different detection techniques. For example, labeling with an AfBP followed by attachment of a fluorescent tag allows experiments such as fluorescence-activated cell sorting (FACS), confocal microscopy, and sodium dodecyl sulfate polyacrylamide gel electrophoresis (SDS-PAGE), whereas attaching a biotin tag allows for affinity purification and proteomics experiments, as illustrated for GPCRs by, e.g., Soethoudt et al. and Beerkens et al.^9,14^ Consequently, only one probe is needed to answer a wide variety of research questions. In this research, this approach was explored for CC chemokine receptor 2 (CCR2).

CCR2 is a class A GPCR found on leukocytes and is involved in a variety of diseases.^18,19^ CCR2 expression has been suggested to be valuable as a biomarker for, e.g., cancer and atherosclerosis due to its prevalence in these diseases.^20,21^ This receptor has two binding sites that can be targeted by small molecules, of which the intracellular allosteric binding site is gaining attention due to the potential for insurmountable antagonism and improved selectivity by intracellular allosteric ligands.^22,23^ Although CCR2 is an attractive drug target with many targeting opportunities, no drugs have made it to the market. Expanding our knowledge of CCR2's expression and pharmacology could benefit drug development for CCR2-related diseases. Novel tools, such as AfBPs, are needed to facilitate this goal. Recently, a fluorescent ligand based on the intracellular ligand SD-24 has been developed by Toy et al. and validated for its use in bioluminescence resonance energy transfer (BRET) assays.^24^ This ligand was used in end-point and kinetic binding assays and was applied in ligand screens. However, because of its fixed fluorescent moiety, the use of this ligand is fairly restricted. Hence, we aimed to provide a more flexible tool compound for CCR2 that can be used in a range of different assays. In this work, we show the development of a CCR2-targeting AfBP and the use of this probe in various assays, such as SDS-PAGE with a conjugated fluorophore, and proteomics experiments with a clicked biotin moiety. Overall, the probe presented in this study is selective within our chosen perimeter and is suitable for a spectrum of applications.

## Results and Discussion

### Probe Design

Rational design started from the previously reported compound **1**, an intracellular covalent antagonist that was previously discovered in our lab upon screening various electrophile-bearing derivatives of SD-24 (Figure 1A, Table 1).^25^ The ability of compound **1** to covalently bind to the intracellular pocket provided an excellent basis for an affinity-based probe as direct competition with orthosteric ligands is avoided.^22,23^ Compound **1** interacts with CCR2’s intracellular binding site via a thiocyanate warhead, likely resulting in a disulfide bridge, which may be reduced by often used strong reduction agents such as β-mercaptoethanol and dithiothreitol, thus potentially limiting its use in chemical biology experiments. To address this potential shortcoming of the electrophilic warhead, additional probes were designed bearing an acrylamide warhead, which provides a more stable covalent linkage as it results in a thioether when reacting with a cysteine residue.^26^

**Table 1:**
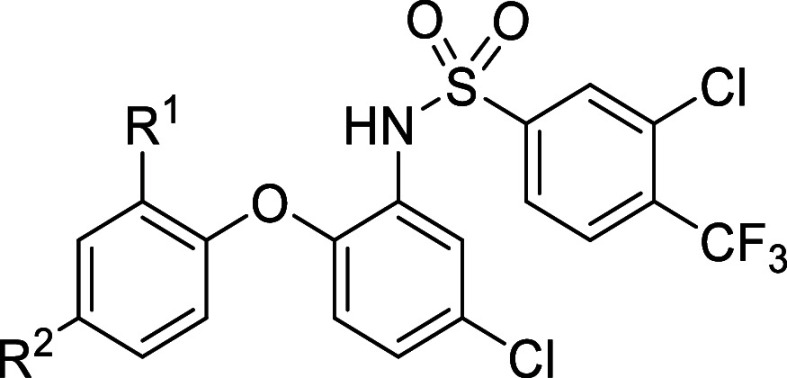

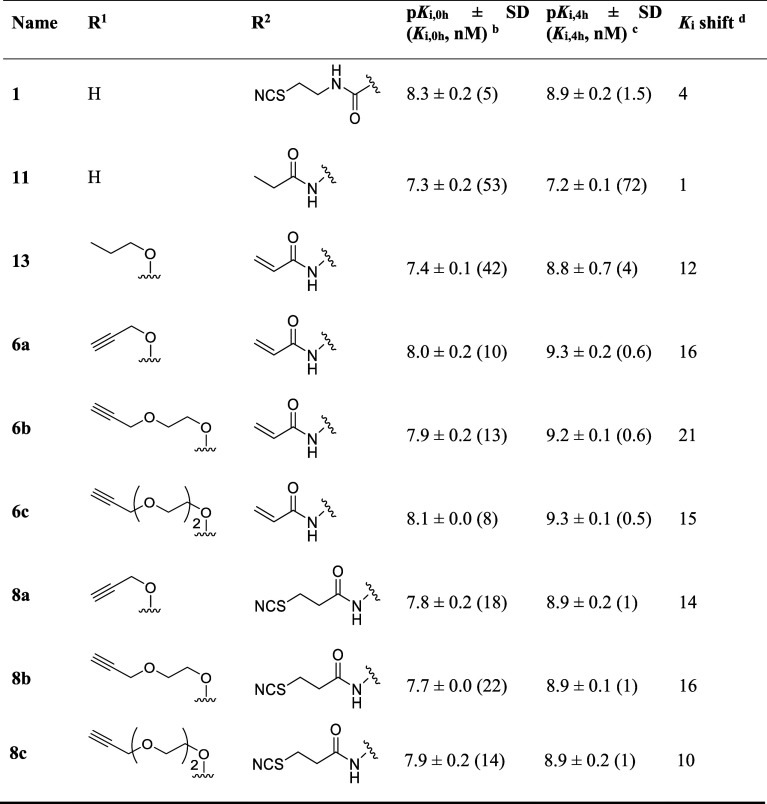
Determination of Time-Dependent Apparent Affinity of Synthesized Probes through [^3^H]CCR2-RA-[*R*] Displacement Assays^a^

a Data are represented as mean ± SD of three experiments performed in duplicate.

b Apparent affinity of probes after 20 min coincubation with [^3^H]CCR2-RA-[*R*].

c Apparent affinity of probes after 4 h preincubation followed by 20 min coincubation with [^3^H]CCR2-RA-[*R*].

d Ratio of mean *K*_i,0h_/*K*_i,4h_.

Next, the optimal incorporation of a ligation handle to facilitate the linkage to a reporter tag was examined. Several ligation strategies are known to achieve this, although bio-orthogonal copper(I)-catalyzed azide–alkyne [3 + 2] cycloaddition is mostly favored due to its high selectivity and reaction rate.^27−29^ Despite the relatively small size of a terminal alkyne, appropriate placement on the intended probe molecule is critical to maintain high target affinity and enable “click” conjugation without steric hindrance from residues within the binding pocket of CCR2. Substitution on the carboxylic acid at the *ortho*-position of the left-hand phenyl ring with a more polar amide was tolerated,^30^ and covalent docking by Ortiz Zacarías et al. of compound **1** revealed this location to be solvent accessible.^25^ Hence, this position was chosen to introduce small ether-based linkers of several lengths containing a terminal alkyne to explore click handle accessibility.

### Synthesis of the Prospective Probes

**Scheme 1:**
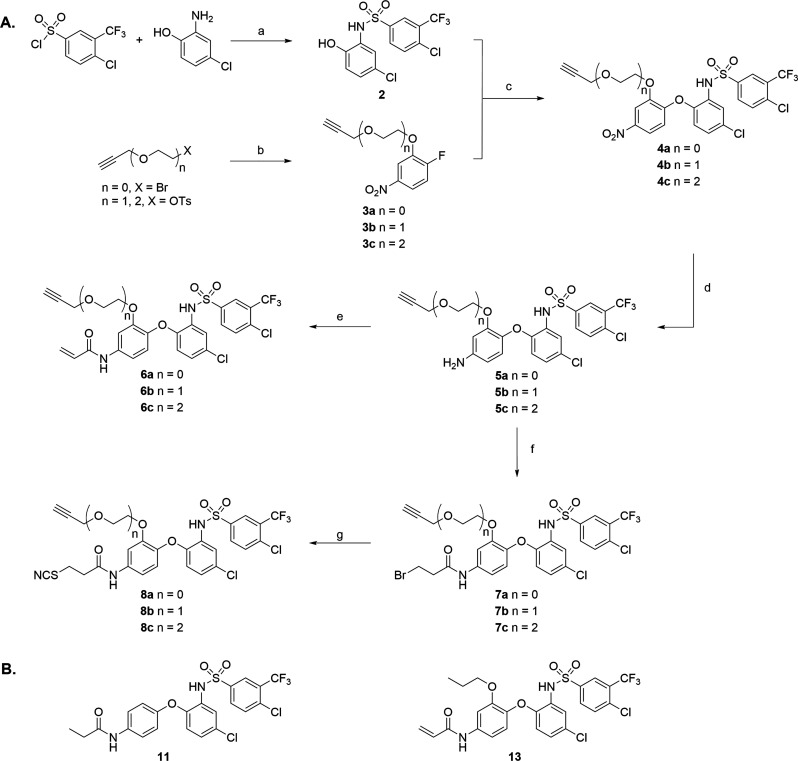
Synthesis of Compounds **6a**–**c** and **8a**–**c** Synthesis of the prospective probs. (A) Reagents and conditions: (a) 4-dimethylaminopyridine, pyridine, reflux, 6 h, 70%; (b) 2-fluoro-5-nitrophenol, K_2_CO_3_, KI, dimethyl sulfoxide, 60 °C, 5 h, 50–55%; (c) K_2_CO_3_, dimethyl sulfoxide, 60–70 °C, 16–48 h, 56–69%; (d) Sn(II)Cl_2_·2H_2_O, ethyl acetate, 60 °C, 20–72 h, 71–80%; (e) acryloyl chloride, dichloromethane, 0 °C, 20–120 min 17–37%; (f) bromopropionyl chloride, ethyl acetate, H_2_O, RT, 20 min–5 h 77–95%; and (h) KSCN, ethanol, reflux, 23–48 h, 53–82%. (B) Molecular structures of control compounds **11** and **13**.

The synthesis of the prospective probes is outlined in Scheme 1A. Briefly, standard DMAP/pyridine conditions provided sulfonamide **2** in good yields.^30^ Separately, O-tosylation of the alkyne-bearing spacers followed by S_N_2-mediated substitution using 2-fluoro-5-nitrophenol afforded intermediates **3a**–**c**. The scaffold was then assembled via S_N_Ar-mediated substitution of the aromatic fluoride of compound **2** with phenolic compounds **3a**–**c**, providing **4a**–**c** in good yields. With the ligation handle installed on the sulfonamide scaffold, we proceeded with the incorporation of the electrophilic warheads. To this end, the nitro group was reduced using tin chloride^15^ to liberate the aniline that was subsequently reacted with either acryloyl chloride (compounds **6a**–**c**) or bromopropionyl chloride (compounds **7a**–**c**). Lastly, the bromide was substituted with potassium thiocyanate to yield the set of thiocyanate-bearing probes **8a**–**c**.

To adequately evaluate compounds **6a**–**c** and **8a**–**c** for their ability to form covalent interactions with CCR2, including an appropriate control compound is critical. Despite the existence of a reversibly binding representative of the sulfonamide scaffold, i.e. SD-24, structural dissimilarities, such as the para-trifluoromethyl instead of the chloride on the right-hand phenyl, and the introduction of a substitution on the para-position of the left-hand phenyl challenge its use as an appropriate control compound. Therefore, sulfonamide derivatives **11** and **13** (Scheme 1B) were synthesized as control compounds (Supplementary Scheme 1).

### Determination of Covalent Binding

**Figure 2:**
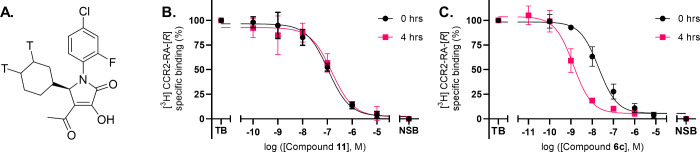
Determination of the apparent affinity of compounds **11** and **6c**. (A) Molecular structure of the radioligand [^3^H]CCR2-RA-[*R*]. (B, C) Time-dependent binding of compounds **11** (B) and **6c** (C) to membranes of U2OS cells stably expressing CCR2 without (0 h) or with (4 h) preincubation followed by coincubation for 20 min with [^3^H]CCR2-RA-[*R*]. Data are represented as mean ± SD of three separate experiments performed in duplicate.

Irreversible binding of the synthesized probes was determined with the so-called *K*_i_ shift assay. In short, U2OS membranes stably expressing CCR2 were either preincubated for 4 h with the compounds or directly coincubated with radioligand [^3^H]CCR2-RA-[*R*] (Figure 2A) for 20 min. The noncovalent control **11** showed overlapping curves; i.e., affinity was not increased by preincubation (Figure 2B). The other probes, such as compound **6c**, showed a leftward shift upon 4 h of preincubation compared to no preincubation (Figure 2C), an indication of covalent binding.^25^ Note that as a dynamic equilibrium cannot be reached between a covalently binding ligand and its target, affinities are reported as “apparent affinity”.^25^

Compound **1** showed a mean apparent affinity of 5 and 1.5 nM without and after 4 h preincubation, respectively, which resulted in a 4-fold shift, matching previous results.^25^ Overall, determined apparent affinities were very high and highly similar within compound sets. Compounds containing acrylamide warheads (**6a**–**c**) showed a 2-fold increase in apparent *K*_i_ (*K*_i,0h_ ∼10 and *K*_i,4h_ ∼0.5 nM, respectively) compared to those with thiocyanate warheads (**8a**–**c**, *K*_i,0h_ ∼20 and *K*_i,4h_ ∼1.3 nM, respectively) (Table 1). Increasing the linker length of click handle R_1_ had a minimal effect on the apparent affinities. All resulting shifts in apparent affinity were greater than that of the initial compound **1**, indicating that all compounds bind irreversibly.

### Specific Labeling of CCR2 on SDS-PAGE

**Figure 3:**
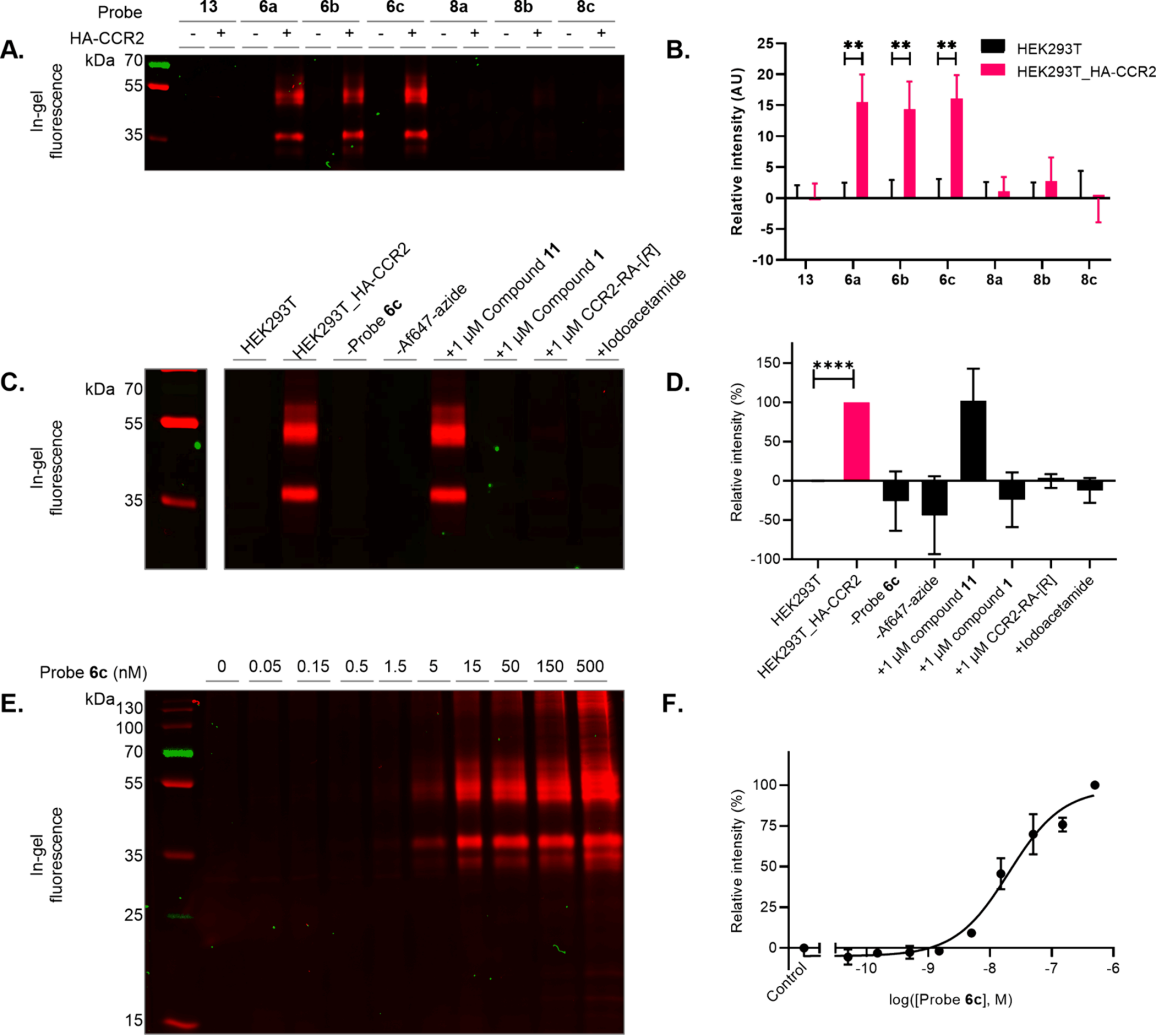
Visualization of HA-CCR2 labeling on SDS-PAGE. (A) In-gel fluorescence of seven probes tested at 100× *K*_i4,h_ with HEK293T membranes (−) or HEK293T membranes transiently expressing HA-CCR2 (+). (B) Quantification of band at ∼54 kDa as a ratio of specific in-gel fluorescence intensity over Coomassie staining, baseline-corrected for HEK293T staining ratio. Images are representative of three separate experiments, and quantification is represented as mean ± SD of three separate experiments. (C) In-gel fluorescence of 50 nM probe **6c** control condition with empty HEK293T cell membranes (HEK293T), HEK293T cell membranes transiently expressing HA-CCR2 (HEK293T_HA-CCR2), without addition of probe **6c** (-probe **6c**) or fluorophore (-Af647-azide), 1 h preincubation with 1 μM of competitors, i.e., compound **11**, compound **1**, or CCR2-RA-[*R*] or cysteine-neutralizing agent iodoacetamide. (D) Quantification of band at ∼54 kDa as ratio of in-gel fluorescence intensity over Coomassie staining. (E) In gel-fluorescence of concentration-dependent labeling of transiently expressed HA-CCR2 in HEK293T cell membranes by probe **6c**. (F) Quantification of band at ∼54 kDa as a ratio of in-gel fluorescence intensity over Coomassie staining. No addition of probe is taken as a control. Images are representative of three separate experiments, and quantification is presented as mean ± SD of three separate experiments. Protein loading controls (Coomassie-stained gels) for the respective figures are shown in Figure S4.

To confirm the prospective probes labeled CCR2, SDS-PAGE experiments were performed with all irreversible ligands in the presence of membranes of HEK293T cells transfected with HA-CCR2 (HEK293T_HA-CCR2, expression confirmed by an enzyme-linked immunosorbent assay (ELISA), Figure S1), which were subsequently conjugated to an Af647 fluorophore for detection (Figure 3). To ensure a similar receptor occupancy for all probes, a concentration corresponding to 100 times the *K*_i,4h_ was used (Figure 3A). Compound **13**, which was shown to likely bind irreversibly but does not contain a click handle, showed no labeling of CCR2 as expected. However, compounds **8a**–**c**, with thiocyanate warheads, also did not show significant labeling. This confirmed our hypothesis of a formed disulfide bridge that was potentially reduced by β-mercaptoethanol present in the denaturation buffer. Thus, the irreversible nature of the probes is critical for receptor labeling, and hence, thiocyanate warheads are unsuitable for this experimental setup.

Whereas probes with thiocyanate warheads were ineffective in labeling CCR2, two protein bands appear at ∼54 and ∼35 kDa for probes **6**–**c**, which were nonexistent in lanes without the receptor (Figure 3A). According to the DNA sequence of HA-tagged CCR2, the expected size of the protein based on its amino acid sequence is ∼46 kDa. As a membrane protein, the actual size can be significantly larger due to post-translational modification (PTM) of CCR2 (see also later in the Results and Discussion section), which may correspond to the upper protein band seen on the gel. This indicates that the probes with an acrylamide warhead were successful in labeling the receptor. Studies with an AfBP for the adenosine A_2A_ receptor showed similar findings in SDS-PAGE experiments with transiently expressed HEK293 cell membranes.^7^

In addition to the expected protein at ∼50 kDa, an additionally labeled protein at ∼35 kDa was observed in both SDS-PAGE and Western blot experiments (Figure 3A), hinting toward the proteolytic cleavage of the CCR2. Hence, we preincubated the samples with an EDTA-free protease-inhibitor cocktail (Figure S2), which revealed no differences in the apparent molecular weights of the labeled proteins on the gel, showing the absence of protease activity on the CCR2 for most common proteases. Moreover, we performed Western blot experiments using an anti-CCR2 antibody. Two protein bands at similar molecular weights were only observed for the transfected cell line, while being absent for the nontransfected cell line, corresponding to the findings from the SDS-PAGE experiments (Figure S3). Taken together, it appears that both bands correspond to different forms of the receptor with different molecular weights. In that sense, the lower-molecular-weight protein could correspond to a truncated version of CCR2, present in the membrane fraction before the execution of the experiment.

For quantification purposes, the higher-molecular-weight protein was assumed to represent the full-length receptor. Therefore, its relative intensity compared to the Coomassie staining was determined as a measure of receptor labeling (Figure 3B). All acrylamide-warhead probes (**6a**–**c**) showed significant labeling compared to untransfected membranes and are strikingly similar in their labeling capacity. Based on its marginally higher apparent affinity and somewhat increased labeling, probe **6c** was chosen for further experiments.

### Characterization of Probe **6c** on SDS-PAGE

To expand the characterization of probe **6c**, several experiments were performed with the same SDS-PAGE setup (Figure 3C–F). First, specific labeling of probe **6c** was determined in HEK293T_HA-CCR2 membranes. As seen before, probe **6c** showed no background labeling in membranes that did not express HA-CCR2 and showed specific receptor labeling at ∼52 and ∼38 kDa in HA-CCR2-expressing membranes (Figure 3C). Quantification of the upper band showed significant labeling compared to the untransfected membranes (Figure 3D). Labeling was absent when HEK293T_HA-CCR2 membranes were incubated without probe **6c** or Af647-azide in the click reaction step, showing no off-target labeling by the fluorophore itself. Preincubation with noncovalent compound **11** followed by probe **6c** incubation showed receptor labeling at the same heights and no significant difference in band intensity as control (101 ± 41%) (Figure 3D). This could be attributable to their markedly different apparent affinity, i.e., 144-fold difference in *K*_i_ after 4 h preincubation. One micromolar of compound **11** is equal to approximately 14 times the *K*_i,4h_, which corresponds to approximately 80–85% receptor occupancy, allowing the remaining 15–20% to be bound by probe **6c**. In addition, as mentioned above in Determination of Covalent Binding, no dynamic equilibrium is reached due to the covalent binding of probe **6c**. Accordingly, compound **11** is displaced more over time, and this balance is apparently quite sensitive, as shown by the considerable variation seen only for this condition enhanced by the relative qualitative character in these types of experiments. For reference, similar chemical biology experiments also showed partial competition on cannabinoid CB_2_R, which was also related to potency disparities.^14^ Covalent compound **1** and CCR2-RA-[*R*] fully competed with probe **6c**, and both bands disappeared entirely after preincubation with either of the compounds. Both **1** and **6c** are high-affinity intracellularly binding compounds, which confirm that probe **6c** binds in the same manner.^23,25^ In addition, preincubation with iodoacetamide, an alkylating agent that binds covalently to the thiol group of cysteines, also prevented receptor labeling. In a previous work, we found either one of three cysteines (C70, C75, or C232) near the intracellular binding pocket to be responsible for the covalent anchoring of ligand **1**. To investigate the involvement of these presumable nucleophilic cysteines in the binding of probe **6a**, *K*_i_ shift experiments with the CCR2^C70S/C75S/C232S^ mutant were performed(Figure S5).^25^ The noncovalent compound **11** showed no shift in apparent affinity for both the WT and the mutant receptor, whereas probe **6c** lost its pronounced shift on the mutant receptor. This supports the idea that probe **6c** not only binds in the same location as compound **1** but also makes use of the same target residue(s) as reported previously.^25^

HEK293T membranes transfected with HA-CCR2 were then incubated with increasing concentrations of probe **6c** (Figure 3E). Quantification of the protein band at ∼52 kDa showed a concentration-dependent increase in labeling, resulting in a pEC_50_ of 7.7 ± 0.2 (Figure 3F), a 44-fold change compared to the apparent affinity that was measured in the *K*_i_ shift assay. Faint labeling may already be observed at 5 nM, but in-gel fluorescence is most visible between 15 and 150 nM, corresponding to 300 times the apparent *K*_i_ at 4 h preincubation. Only at 500 nM (1000× apparent *K*_i,4h_) is labeling observed at ∼130, 60, 16, and 18 kDa, which could be either aggregates of the receptor (labeling at ∼130 kDa) or unspecific protein labeling due to the high concentration of the probe.

### Probe **6c** as a Tool in Diverse SDS-PAGE Experiments

**Figure 4:**
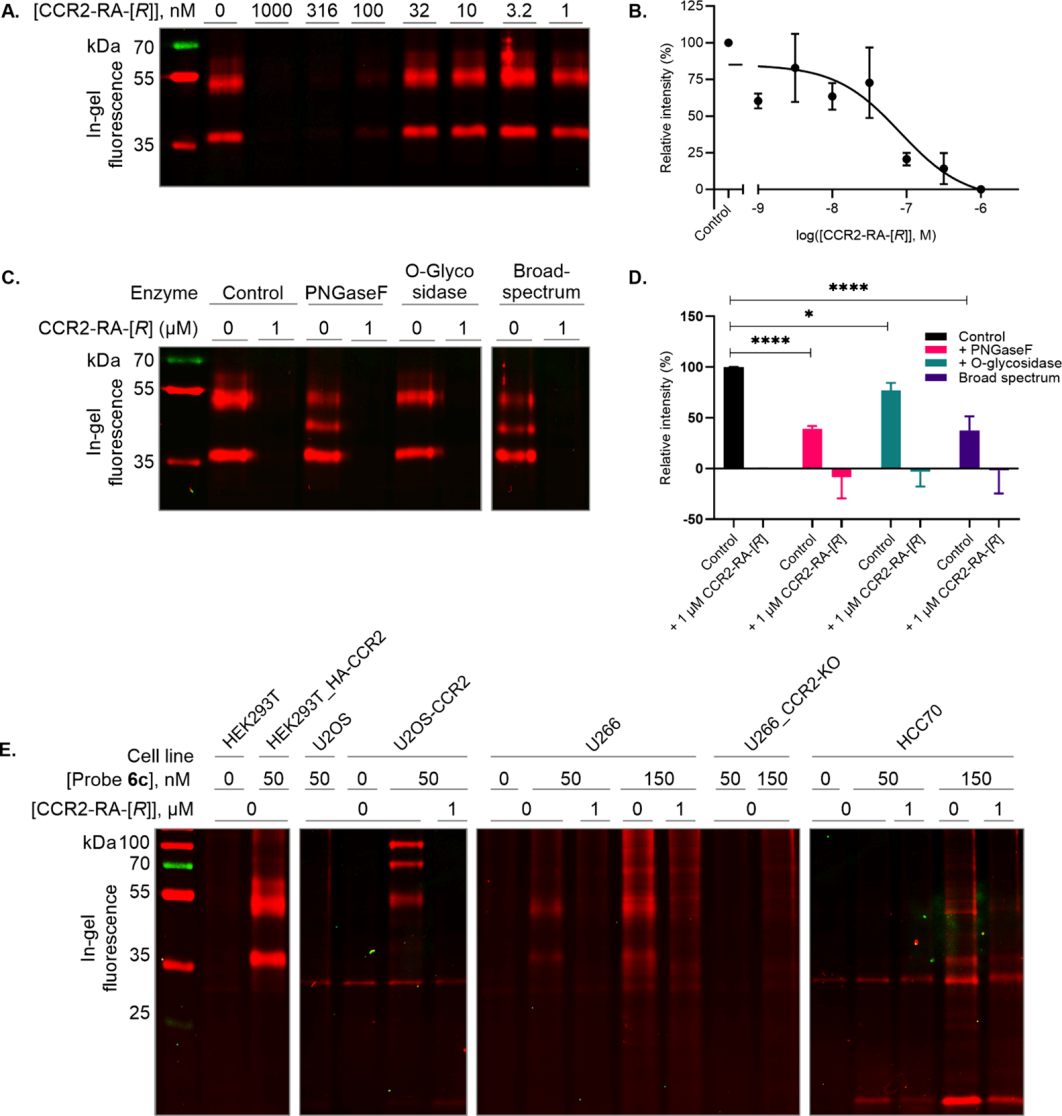
Application of probe **6c** in diverse SDS-PAGE-based experiments. (A) In-gel fluorescence of concentration-dependent inhibition by CCR2-RA-[*R*] preincubated with HEK293T cell membranes transiently expressing HA-CCR2 for 1 h before labeling with 50 nM probe **6c**. (B) Quantification of the protein band at ∼54 kDa as the ratio of in-gel fluorescence intensity over Coomassie staining. (C) Detection of post-translational modification in HEK293T cell membranes transiently expressing HA-CCR2 by preincubation with PNGaseF, *O*-glycosidase, and a broad-spectrum mix of post-translational modification enzymes with or without 1 μM CCR2-RA-[*R*] before labeling with 50 nM probe **6c**. (D) Quantification of the protein band at ∼54 kDa as the ratio of in-gel fluorescence intensity over Coomassie staining. (E) In-gel fluorescence of labeling of CCR2 in different cell lines by probe **6c**. Nontransfected HEK293T cell membranes (HEK293T), HEK293T cell membranes transiently expressing HA-CCR2 (HEK293T_HA-CCR2), nontransfected U2OS cell membranes (U2OS), U2OS cell membranes stably expressing CCR2 (U2OS-CCR2), U266 B-lymphocyte cell membranes (U266), CRISPR-Cas9-generated CCR2-KO U266 (U266_CCR2-KO), and HCC70 breast cancer cell line (HCC70) preincubated with or without 1 μM CCR2-RA-[*R*] for 1 h before labeling with 0, 50, or 150 nM probe **6c**. Images are representative of three separate experiments, and quantification is presented as mean ± SD of three separate experiments. Protein loading controls (Coomassie-stained gels) for the respective figures are shown in Figure S4.

To further establish the versatility of probe **6c** in SDS-PAGE experiments, a variety of applications were investigated. First, HEK293T_HA-CCR2 membranes were preincubated with increasing concentrations of CCR2-RA-[*R*] and subsequently treated with 50 nM probe **6c** (Figure 4A) to show whether probe **6c** can interact with CCR2 in such a way that competitive binding of other ligands can be detected. Labeling was completely reversed at 1000 nM CCR2-RA-[*R*], and at 1 nM, the signal was close to control. A concentration–inhibition curve was generated, resulting in an observed pIC_50_ of 7.0 ± 0.4 (Figure 4B). This shows that probe **6c** can indeed be used to visualize binding of competing ligands to the CCR2 receptor and therefore could be used as a tool compound for screening novel ligands.

Second, we examined whether probe **6c** could be used to detect PTMs (Figure 4C). Preincubation with PNGase, which cleaves N-glycans, resulted in an additional protein band at ∼45 kDa, which overlapped with the expected height of HA-CCR2 of ∼46 kDa. In addition, a significant reduction of the upper band was observed after a brief pretreatment with PNGase. Similarly, pretreatment with *O*-glycosidase, which cleaves *O*-glycans, also resulted in a significant decrease in the intensity of the upper band. However, this is likely an artifact of the assay, as detected bands are still visible at the same molecular weight as the control without emergence of additional bands. Lastly, pretreatment with a broad-spectrum enzyme mix containing PNGase, *O*-glycosidase, and α2-3,6,8 neuramidase revealed a new band at ∼45 kDa, a significant reduction of the upper band similar to the PNGase treatment (Figure 4D).

PTMs have a regulatory function in chemokines and their receptors, including a potential role in cancer.^31^ For CCR2, confirmed PTMs include N-linked glycosylation of asparagine 14 in the N-terminus.^32^ The reduction in weight of CCR2 brought on by PNGase was approximately 5 kDa, similar to that seen by Preobrazhensky et al. (2000).^32^ In addition, Verhallen et al. (2023) also showed that CCR2 was not O-glycosylated in transfected CHO cells, confirming that the observed effect of *O*-glycosidase treatment on the intensity of the upper band is likely an artifact.^33^ Furthermore, the exploration of CCR2 PTMs in a variety of cellular backgrounds would provide information about the role and regulation of CCR2.

Finally, probe **6c** was tested for its ability to label CCR2 in other relevant cell lines (Figure 4E). In membranes from U2OS cells stably expressing CCR2 (U2OS-CCR2), which were also used for *K*_i_ shift assays, multiple labeled proteins at heights of ∼97, 71, 53, and 38 kDa were observed that were not visible in the untransfected U2OS membranes and were absent when preincubated with CCR2-RA-[*R*]. In the U2OS-CCR2 cell membranes, CCR2 is fused to a TEV protease site and a Gal4-VP16 transcription factor (Invitrogen), which results in a construct of at least 74 kDa, similar to the labeled protein observed at ∼71 kDa. The protein at ∼31 kDa, which was visible for all lanes even in the absence of probe **6c**, appears to be nonspecific labeling of the fluorophore. Moreover, two human cancer cell lines, namely, U266 and HCC70, were investigated with reported endogenous expression of CCR2, where first CCR2 expression was confirmed by specific [^3^H]-CCR2-RA-[*R*] binding (Figure S6).^34^ In U266 membranes, a human monocytic cell line, protein labeling was observed at ∼67 and 57 kDa. Here, the in-gel fluorescence intensity appears to be concentration-dependent, although this may be attributed to increased nonspecific labeling. In addition, U266 cells with a CCR2 knockout (U266_CCR2-KO) were generated using CRISPR-Cas9 editing technology in which binding of [^3^H]INCB3344 was successfully reduced compared to wild-type cells (Figure S7). On SDS-PAGE, labeling completely disappeared when U266_CCR2-KO membranes were incubated with 50 nM probe **6c.** Moreover, when using 150 nM probe **6c**, labeling was markedly decreased. In HCC70, a human triple-negative breast cancer cell line, protein labeling appeared at ∼49, 36, and 17 kDa. Although only visible at high concentrations of probe **6c** (150 nM), the fluorescence intensity was markedly reduced for bands at ∼49 and 36 kDa by preincubation with the competing CCR2 antagonist CCR2-RA-[*R*], indicative of specific receptor labeling. However, the protein band at ∼17 kDa represented nonspecific labeling of the probe. Interestingly, the variety in molecular weight of CCR2 for each cell type could validate the heterogeneous expression of CCR2 on the cell membrane such as CCR2 modified with PTMs. All in all, these results substantiate the utility of probe **6c** in labeling CCR2 in a variety of cell lines, including those that endogenously express (low levels of) CCR2 (U266, HCC70).

### Detection of CCR2 Using Mass-Spectrometry-Based Proteomics

Thus far, we have shown that probe **6c** is a versatile tool for its use in SDS-PAGE experiments. However, to exploit the versatility of this probe to its fullest, other detection moieties instead of only fluorophores can be added.^35^ An azide-tagged biotin moiety was clicked to probe **6c** instead to investigate whether this probe could also be used in mass-spectrometry-based pull-down experiments. First, CCR2 was irreversibly labeled by probe **6c** followed by a click reaction with biotin-azide, pull down using avidin-coated agarose beads, protein digestion by chymotrypsin, and subsequent mass spectrometry analysis.

**Figure 5:**
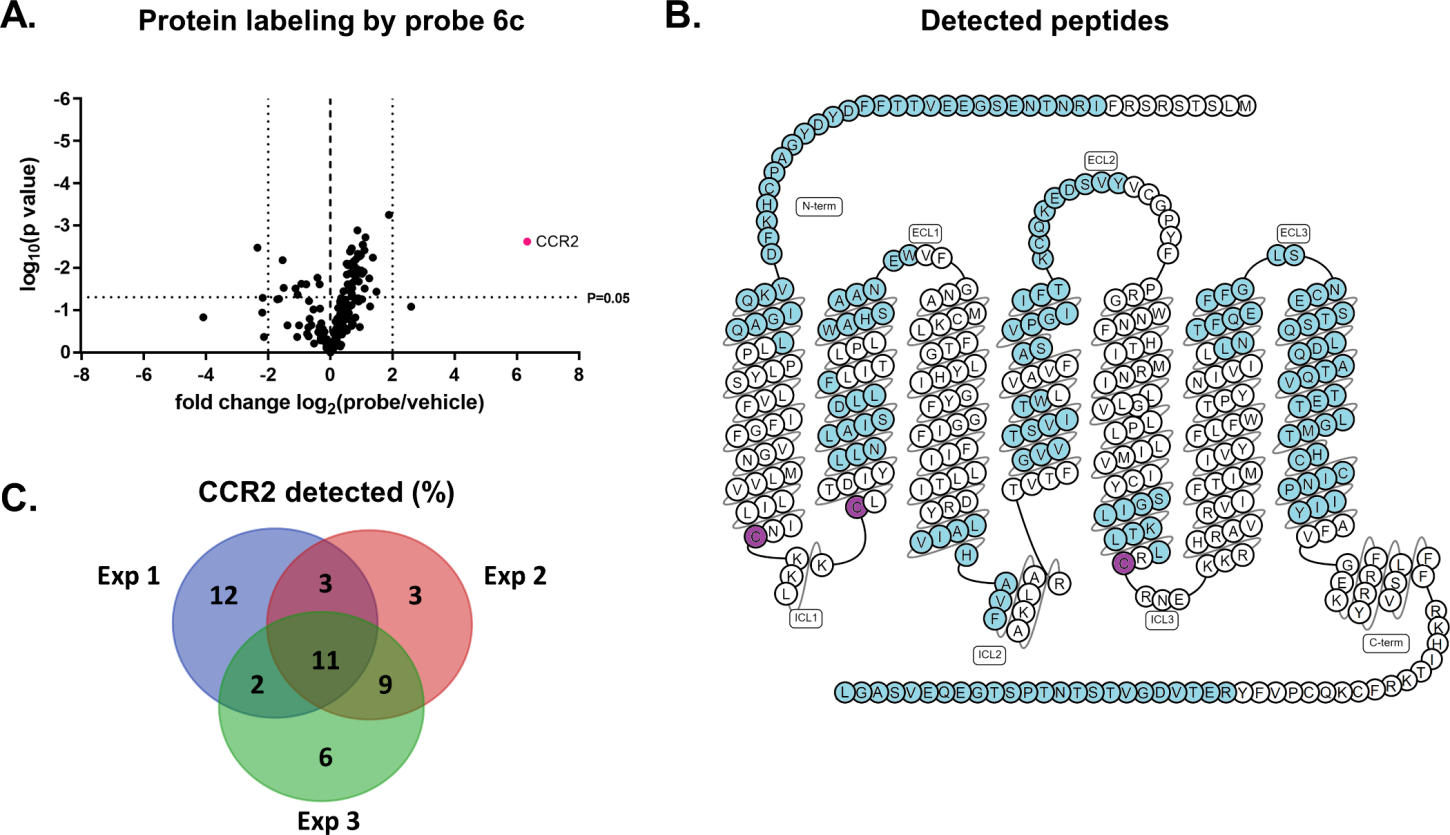
Application of probe **6c** in affinity-based pull-down proteomics. (A) Exemplary volcano plot of samples containing HEK293T_HA-CCR2 membranes treated with 1 μM probe **6c** or the vehicle control (1% DMSO). Data are plotted as enrichment ratio (log_2_(ratio) and probability (−log_10_(*p*)) as determined in a multiple *t* test. The dotted lines indicate threshold values of a ratio > 2 and a *p* value < 0.05. The experiment was performed in triplicate. (B) Venn diagram showing a comparison of the percentage of detected CCR2 amino acid residues between three different pull-down experiments. All experiments were performed in triplicate. (C) Snake plot of CCR2 with the recovered peptides highlighted in blue and proposed targeted cysteine residues in purple (adapted from GPCRdb.org). Peptides were detected in either of the performed experiments.

During all of the performed experiments (*n* = 3), CCR2 was found to be highly enriched as compared to vehicle-treated samples (Figure 5A), showing a high fold change. Compared to the other detected proteins, the CCR2 showed a >10 times higher fold change. This fold change could be reduced by preincubation with the irreversible CCR2 antagonist compound **1** (Figure S8), indicating that the detected signal comes from specific binding of the probe to the intracellular binding pocket of the CCR2. Although, when the performed experiments were combined, a sequence coverage of 46% of the CCR2 was detected (Figure 5B), there was a variation in the amount and size of the peptides detected (Figure 5C). Only 11% of the receptor was detected consistently between experiments, presumably caused by the use of chymotrypsin as a protease, which has been found to often miss potential cleavage sites.^36^ Of note, no peptides containing the predicted interacting cysteines (i.e., C70, C75, and C232) were recovered, as those were still bound by the probe and biotin and thus removed in final purification steps when the beads were removed from the samples.

Taken together, probe **6c** showed a high enrichment of the CCR2 upon pulling down the receptor from transfected HEK293T membranes. Having such an assay setup allows for further applications of **6c** in proteomics experiments to, e.g., study the role of PTMs and protein–protein interactions, analogous to enzyme-targeting probes.^37^

## Conclusions

In this work, we synthesized and validated probe **6c** as the first intracellular allosteric-affinity-based probe targeting CC chemokine receptor 2. This probe showed remarkable versatility due to the incorporation of a click handle, allowing the probe to be used in both SDS-PAGE and mass-spectrometry-based proteomics depending on the reporter tag (fluorescent or biotin) attached to the probe using click chemistry. Furthermore, probe **6c** showed high specificity in both assay setups.

As there are currently no drugs on the market that target the CCR2 receptor, novel tools and strategies are needed to aid and promote drug discovery efforts. In the current manuscript, we have specifically touched upon the utilization of affinity-based probes to (i) determine the affinity of a known intracellular CCR2 binder (CCR2-RA-[*R*]) and (ii) explore the expression of CCR2 in a variety of cell lines known to express CCR2. Such experiments could be expanded toward the screening of novel ligands targeting the intracellular binding site of CCR2, as well as screening cells on the presence of CCR2 to promote rational targeting of receptor populations in certain pathologies. Probe **6c** therefore represents a valuable alternative to traditional tool compounds such as primary antibodies and radiolabeled ligands. Thus, a probe like **6c** could aid in the discovery of efficacious therapies against hard-to-target GPCRs such as the CCR2 receptor.

## Supporting Information Available

Supplemental figures and tables, experimental methods, NMR spectra, and HPLC analyses for all tested compounds (PDF)
Pull-down proteomics raw data (XLSX)

## ABBREVIATIONS

ABP: activity-based probe
AfBP: affinity-based probe
BRET: bioluminescence resonance energy transfer
CCR2: CC chemokine receptor 2
CuAAC: copper-catalyzed azide–alkyne cycloadditions
FACS: fluorescence-activated cell sorting
GPCR: G protein-coupled receptor
LC/MS: liquid chromatography/mass spectrometry
SDS-PAGE: sodium dodecyl sulfate polyacrylamide gel electrophoresis
