# Supplementary material for: Labeling of CC Chemokine Receptor 2 with a Versatile Intracellular Allosteric Probe
Source: ACS Chem Biol. 2024 Aug 26;19(9):2070–80. doi: 10.1021/acschembio.4c00439 (PMC11420878; doi:10.1021/acschembio.4c00439)
Supplement: Supplementary file 1 — cb4c00439_si_001.pdf [file cb4c00439_si_001.pdf]

- **Supporting Information –**

Labelling of CC chemokine receptor 2 with a versatile intracellular allosteric probe

L.S. den Hollander[*,1], B.L.H. Beerkens[*,1], S. Dekkers[*,1], J.P.D. van Veldhoven[1], N.V. Ortiz Zacarías[1,2], C. van der Horst[1], E.G. Sieders[1], B. de Valk[1], J. Wang[1], A.P. IJzerman[1], D. van der Es[#,1], L.H. Heitman[#,1,2]

[*,#] authors contributed equally

[1] Leiden Academic Centre for Drug Research, Division of Drug Discovery and Safety, Leiden, the Netherlands

[2] Oncode Institute, Leiden, the Netherlands

Table of contents

Supplementary figures S2
Figure S1 S2
Figure S2 S2
Figure S3 S3
Figure S4 S4
Figure S5 S5
Figure S6 S6
Figure S7 S7
Figure S8 S8
Supplementary schemes S9
Experimental methods S10
Chemistry S10
Biology S18
^{1}H NMR spectra S24
HPLC for purity analyses S28
References S32

Supplementary figures

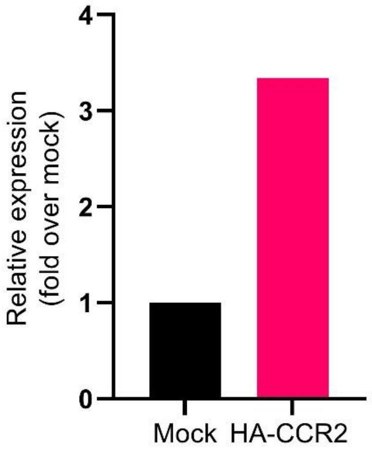

Figure S1. Relative expression of HA-CCR2 transfected into HEK293T cells as measured with ELISA. HEK293T cells were transfected with either 5 µg pcDNA3.1 (Mock) or 5 µg pcDNA3.1_HA-CCR2 (HA-CCR2) plasmids. Expression of HA-CCR2 in HEK293T cells was measured using an anti-HA antibody. Data is shown as a representative graph of at least three separate experiments performed in quintuplicates.

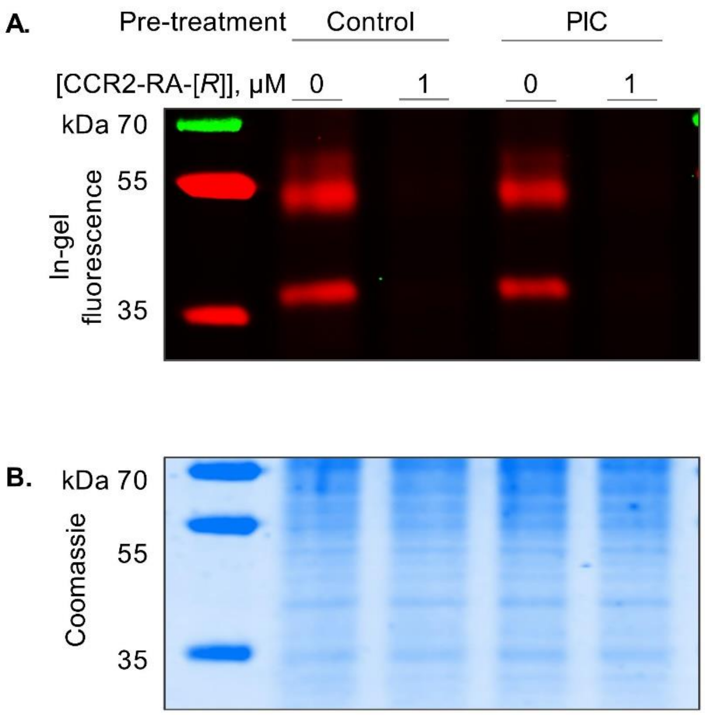

Figure S2. Effect of a protease-inhibitor cocktail (PIC) on HA-CCR2 visualization on SDS-PAGE. (A) HEK293T cell membranes transiently expressing HA-CCR2 were pre-incubated with a protease inhibitor cocktail (PIC) and with or without 1 µM CCR2-RA-[*R*] before labelling with 50 nM probe **6c**. (B) corresponding Coomassie staining. SDS-PAGE and Coomassie staining images are cropped to preserve space.

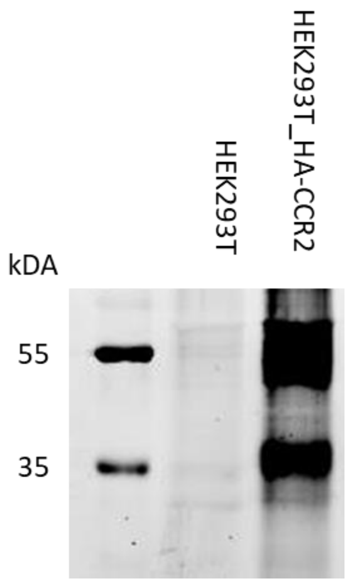

Figure S3. Immunoblotting of CCR2 expression in transiently HA-CCR2 transfected HEK293T cell membranes. Experiments were performed with an anti-CCR2 primary antibody and a Horseradish Peroxidase- (HRP) conjugated secondary antibody, before addition of enhanced chemiluminescence (ECL) reagent. Specific bands were observed around ~35 and ~55 kDa. Image is a representative image of three separate experiments. Image is cropped to preserve space.

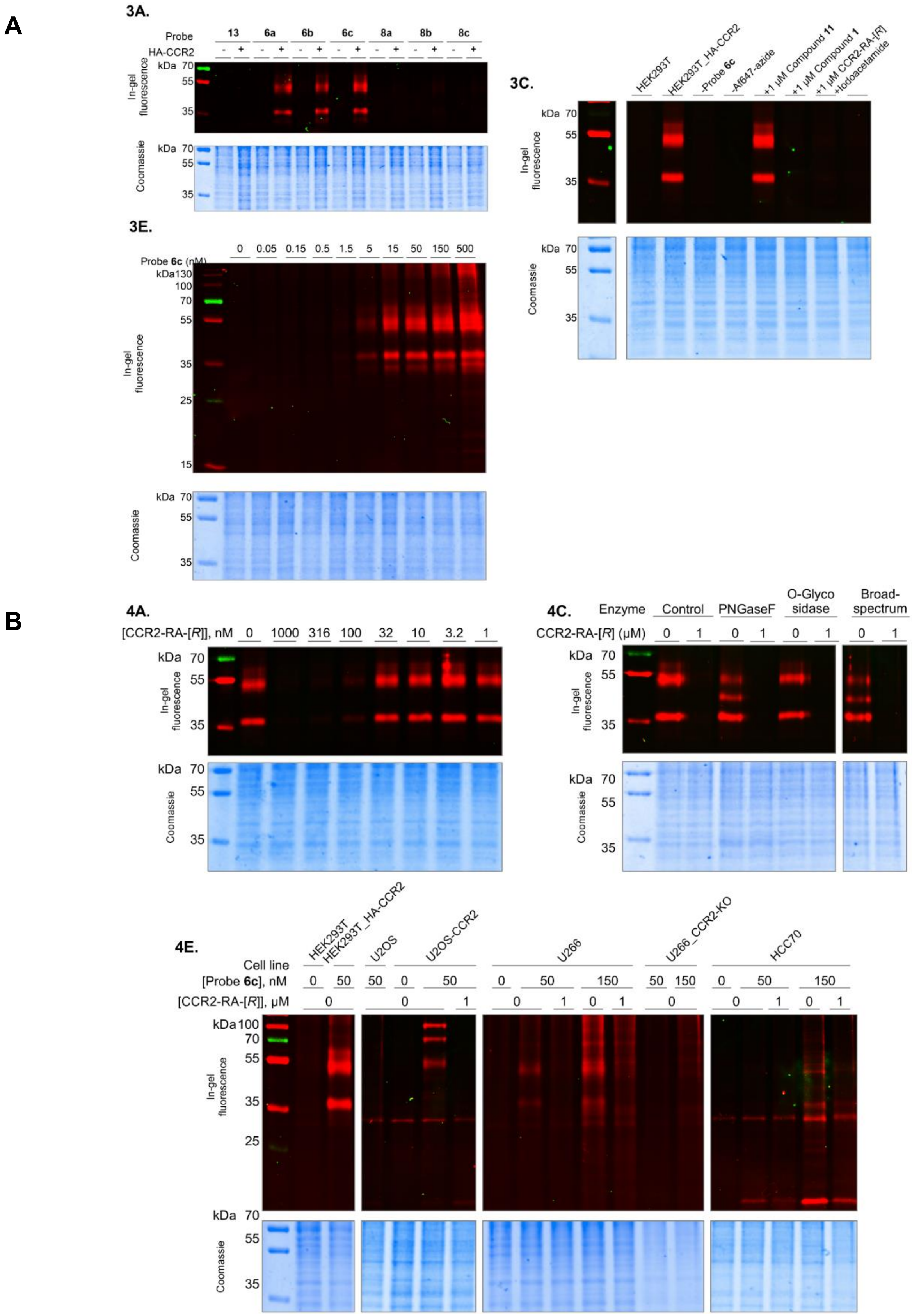

Figure S4. Protein loading controls of the gel images shown in Figures 3 and 4. The imaged gels were stained with Coomassie Brilliant Blue (CBB) overnight, de-stained using (H_2O:MeOH:AcOH 5:4:1) and afterwards imaged using Coomassie settings. Shown are the gel images from Figures 3 (A) and 4 (B) and the respective protein loading controls.

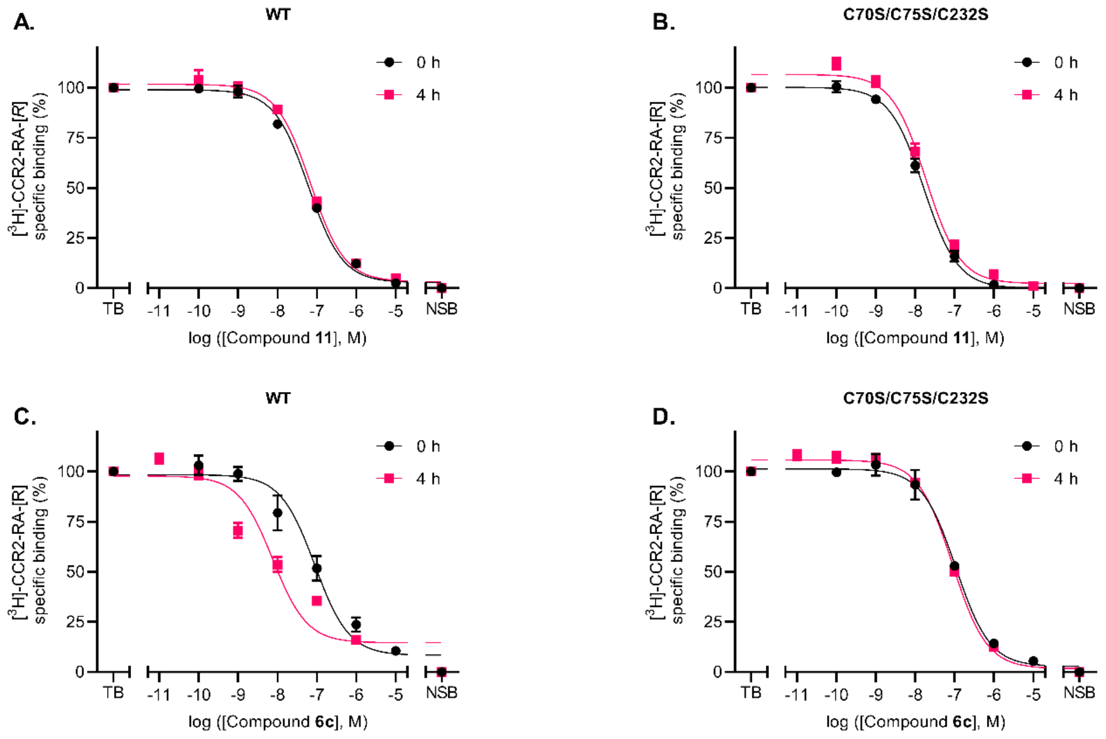

Figure S5. Determination of apparent affinity of compounds 11 and 6c. Time-dependent binding of probes to membranes of CHO cells transiently expressing FLAG-CCR2 (A, C) or FLAG-CCR2-70S/C75S/C232S (B, D) cells either without or with 4 hours pre-incubation followed by co-incubation for 20 minutes with [^{3}H]CCR2-RA-[*R*]. Data is represented as mean ± SD of three separate experiments performed in duplicate.

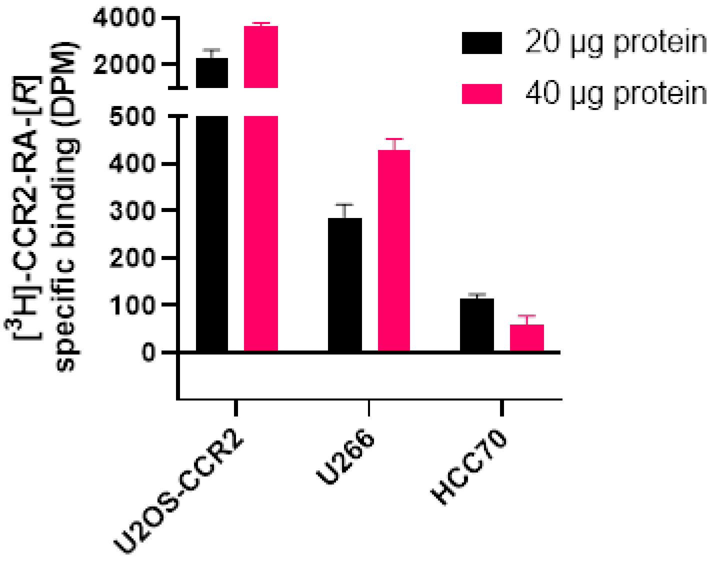

S6. Determination of specific [^{3}H]CCR2-RA-[*R*] binding to U2OS-CCR2, U266 and HCC70 membranes. Specific binding was determined for 20 and 40 µg of protein by subtracting non-specific binding, determined by 10 µM CCR2-RA-[*R*], from the total binding. Data is shown as mean ± SD of two separate experiments performed in duplicate.

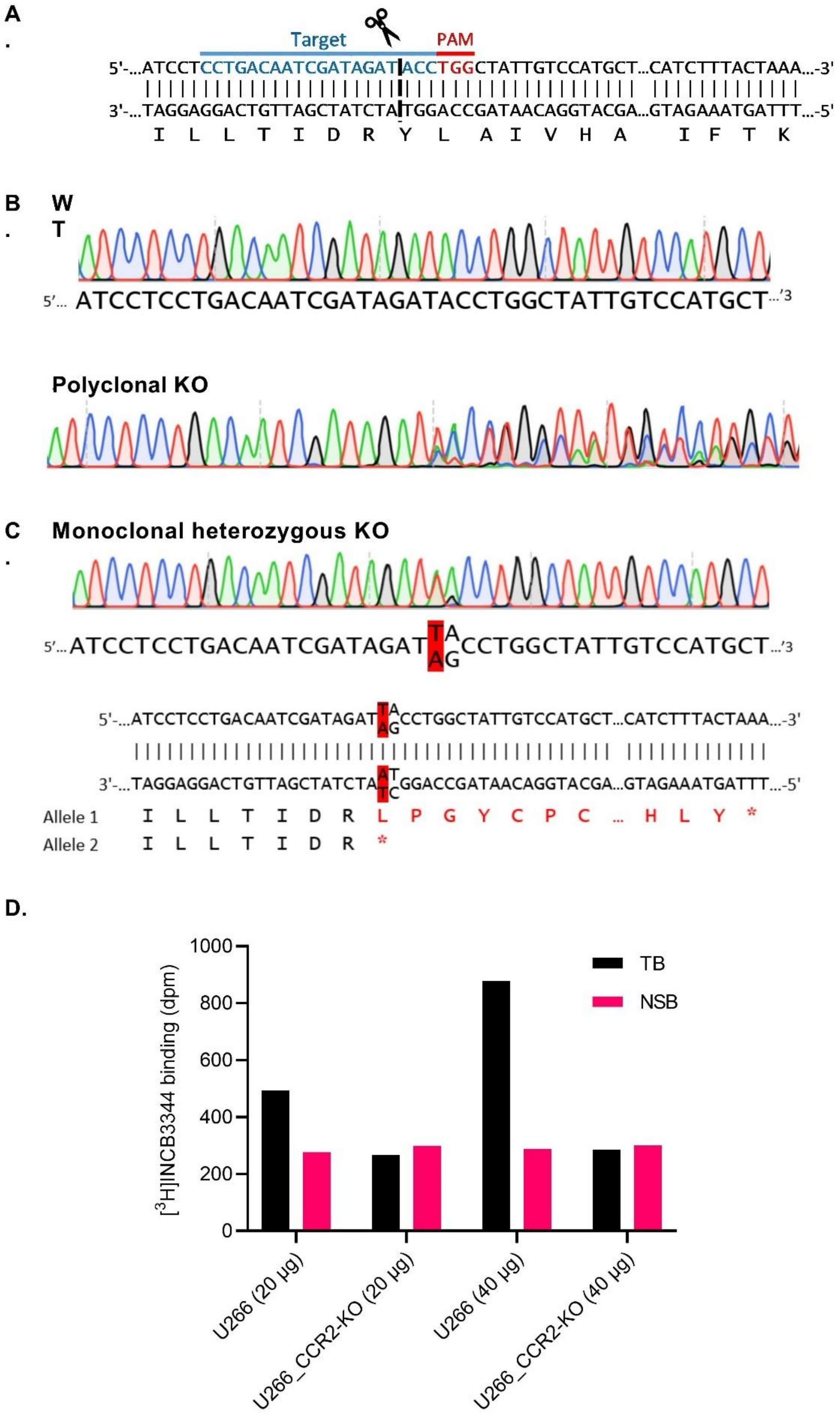

Figure S7. Validation of CRISPR-Cas9 generated CCR2 knock-out U266 cell line. (A) CRISPR-Cas9 aided CCR2 knock out strategy in the U266B1 cell line. The selected guide RNA targets the target site (blue line) resulting in a double-strand break three base pairs upstream of the PAM (red line) site. The corresponding protein translation is also depicted. (B) Sanger sequencing traces of WT and polyclonal

CCR2 KO U266B1 cells. Mixed traces indicate different gene editing events in different alleles in different cells. (C) Sanger sequencing trace of monoclonal heterozygous CCR2 KO U266B1 cell line. Double traces indicate different gene editing events in the different alleles. The corresponding protein translations show early truncated CCR2 caused by a +1 frameshift. (D) Binding of radiolabelled CCR2 antagonist [^{3}H]INCB3344 (~6.5 nM) to U266B1 WT and U266_CCR2-KO membranes (20 and 40 µg) at 25 °C incubated for 2 hours in the absence (Total binding; TB) and presence (Non-specific binding; NSB) of 10 µM CCR2 antagonist BMS CCR2 22. Data is shown as mean of a representative experiment performed in duplicate. Two independent experiments have been performed with similar results.

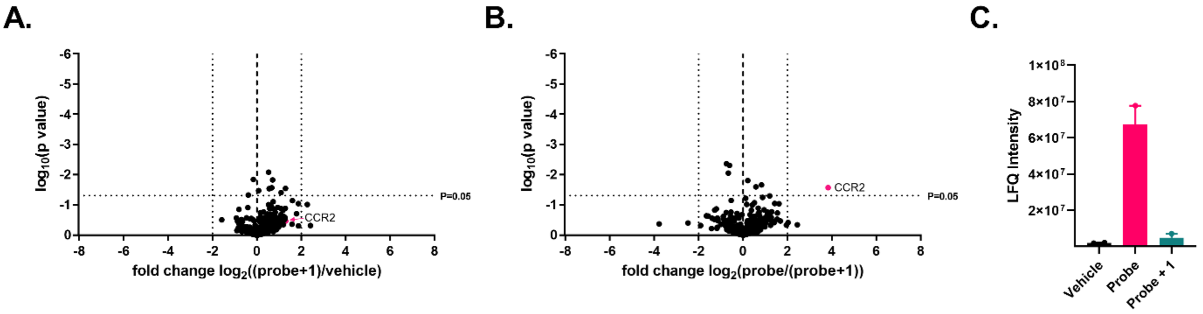

Figure S8. Competition for the CCR2 intracellular binding pocket in proteomic pull-down experiments. Pull-down samples were either pre-treated with or without 10 µM of irreversible antagonist compound **1**, prior to probe incubation, click reaction and further sample preparation. (A) Comparison between the samples that were pre-treated with compound **1** to the vehicle control (1% DMSO) ((probe+**1**)/vehicle). No significant enrichment of the CCR2 was detected after pre-incubation with antagonist **1**. (B) Comparison of the probe-treated samples to the samples that were pre-treated with compound **1** (probe/(probe+**1**)). There is a high enrichment of CCR2 in the positive samples, as compared to the pre-treated samples. (C) Bar graph showing the label free quantification (LFQ) intensity of the CCR2 in the three conditions: vehicle control (1% DMSO), probe-treated and pre-treated with compound **1**. Volcano plot data is plotted as enrichment ratio ($\log_2$(Ratio)) and probability ($-\log_{10}$(p))as determined in a multiple t test. Data originates from N=2 containing triplicates. The dotted lines indicate threshold values of a ratio > 2 and a p-value < 0.05.

Supplementary schemes

Scheme S1. Synthesis of compounds 11 and 13

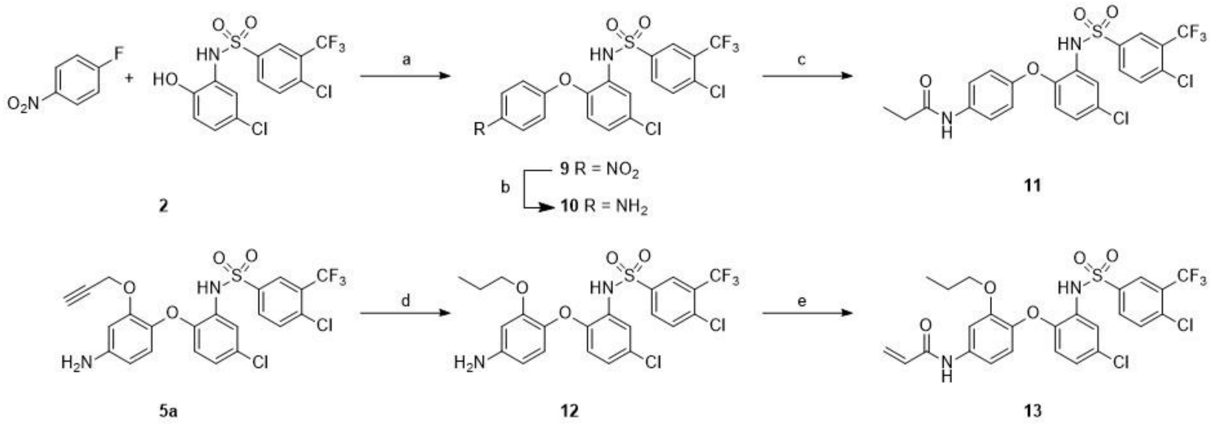

Reagents and Conditions: (a) K_2CO_3, dimethyl sulfoxide, 60 °C, 20 h, 56%; (b) Sn(II)Cl_2·$2H_2O$, ethyl acetate, 40 °C, 72 h, quant.; (c) (i) acryloyl chloride, dichloromethane, RT, 18 h, 25%; (ii) Pd/C (10% wt), H_2, methanol, 4 h, 75%; (d) Pd/C (10% wt), H_2, methanol, 3 h, quant.; (e) acryloyl chloride, dichloromethane, RT, 4 h, 16%.

Experimental procedures

Chemistry

General chemistry. *General Methods.* Solvents and reagents were purchased from commercial sources and were of analytical grade. All reactions were monitored with thin layer chromatography (TLC), using aluminium silica gel coated 60 F_{254} plates from Merck. Purification by column chromatography was carried out using VWR silica gel (60–200 µm). Purification by semi-preparative HPLC was performed on a Shimadzu HPLC system running a Phenomenex Gemini® C18 110 Å column (100 x 10.0 mm x 5 µm). Sample elution time was 15 minutes at a flowrate of 7.0 mL/min, using a gradient of 90:10:0.1% H_2O/CH_3CN/formic acid to 10:90:0.1% H_2O/CH_3CN/formic acid. Nuclear magnetic resonance (NMR) spectra were recorded on either a Bruker AV-400 liquid spectrometer (^1H NMR, 400 MHz) or on a Bruker DPX-300 (^1H NMR, 300 MHz) at ambient temperature and subsequently analyzed with MestReNova v.14 software. Chemical shifts are reported in parts per million (ppm), designated by δ and corrected to the chemical shift of the deuterated solvent. Coupling constants (*J*) are reported in hertz and the signal multiplicities are described by the following: s, singlet; br s, broad singlet; d, doublet; t, triplet; q, quartet; m, multiplet; dd, doublet of doublets. Identity and analytical purity of the synthesized compounds were determined using liquid chromatography mass spectrometry (LC-MS) using a Shimadzu HPLC system running a Phenomenex Gemini® C18 110 Å column (50 mm × 4.6 mm × 3 µm) and coupled to a Shimadzu LCMS-2020 Single Quadrupole Mass Spectrometer. Sample elution time was 15 minutes at a flowrate of 0.55 mL/min, using a gradient of 90:10:0.1% H_2O/CH_3CN/formic acid to 10:90:0.1% H_2O/CH_3CN/formic acid. All compounds are >95% pure by HPLC.

*4-Chloro-N-(5-chloro-2-hydroxyphenyl)-3-(trifluoromethyl)benzenesulfonamide (**2**).* Commercially available 4-chloro-3-(trifluoromethyl)benzenesulfonyl chloride (1.33 g, 4.75 mmol) and 2-amino-4-chlorophenol (0.62 g, 4.32 mmol) were taken up in pyridine (25 mL) and to it was then added 4-dimethylaminopyridine (0.05 g, 0.43 mmol, 10 mol%). The resulted solution was stirred at a reflux for 5 hours after which TLC indicated full consumption of 2-amino-4-chlorophenol. The reaction mixture was concentrated *in vacuo*, diluted with ethyl acetate and subsequently washed with 1N HCl (3x) and brine. The organics were dried over $MgSO_4$ and the crude was purified by column chromatography, eluting with 40% ethyl acetate in petroleum ether. Recrystallization from hexanes afforded the title compound as off-white crystals. Yield: 70% (1.17 g, 3.0 mmol). ^1H NMR (400 MHz, $CDCl_3$) δ 8.08 (d, *J* = 2.2 Hz, 1H), 7.83 (dd, *J* = 8.4, 2.3 Hz, 1H), 7.61 (d, *J* = 8.4 Hz, 1H), 7.20 (d, *J* = 2.4 Hz, 1H), 7.05 (dd, *J* = 8.6, 2.5 Hz, 1H), 6.84 (br s, 1H), 6.77 (d, *J* = 8.6 Hz, 1H), 5.79 (s, 1H).

*1-Fluoro-4-nitro-2-(prop-2-yn-1-yloxy)benzene (**3a**).* Commercially available 2-fluoro-5-nitrophenol (0.63 g, 4.0 mmol) and propargyl bromine (in 80% toluene, 0.48 g, 4.0 mmol) were taken up in acetone (25 mL) and to it was then added K_2CO_3 (1.38 g, 10.0 mmol, 2.5 equiv.). The resulted suspension was stirred at 40°C for 16 hours, after which the reaction mixture was diluted with ethyl acetate subsequently washed with 1N HCl (1x), H_2O (1x) and brine (1x). The organics were dried over $MgSO_4$, concentrated *in vacuo* and ultimately purified by column chromatography, eluting with 10% ethyl acetate in petroleum ether yielding the title compound as a yellow solid. Yield: 85% (0.66 g, 3.4 mmol). ^1H NMR (400 MHz, $CDCl_3$) δ 8.03 (dd, *J* = 7.2, 2.7 Hz, 1H), 7.97 – 7.89 (m, 1H), 7.25 (dd, *J* = 9.9, 8.9 Hz, 1H), 4.88 (d, *J* = 2.5 Hz, 2H), 2.64 (t, *J* = 2.4 Hz, 1H).

*1-fluoro-4-nitro-2-(2-(prop-2-yn-1-yloxy)ethoxy)benzene (**3b**).* Step one: To a stirring solution of commercially available 2-(prop-2-yn-1-yloxy)ethan-1-ol (2.0 g, 20.0 mmol) in dichloromethane (25 mL) were added triethylamine (5.58 mL, 40.0 mmol, 2.0 equiv.) and 4-dimethylaminopyridine (0.24 g, 2.0 mmol, 10 mol%). This solution was then cooled to 0 °C after which was added in dropwise manner tosyl chloride (3.81 g, 20.0 mmol, 1 equiv., in 25 mL dichloromethane). After completion of the addition the

reaction mixture was allowed to warm to room temperature at which it was then maintained for 72 hours. Solvents were removed *in vacuo* and the crude product was purified by column chromatography eluting with 10-20% ethyl acetate in petroleum ether as eluent, affording the tosylate as a colourless oil. Yield: 45% (2.25 g, 9.0 mmol). ^{1}H NMR (400 MHz, $CDCl_3$) δ 7.79 (d, J = 8.3 Hz, 2H), 7.35 (d, J = 8.2 Hz, 2H), 4.23 – 4.16 (m, 2H), 4.11 (d, J = 2.4 Hz, 2H), 3.77 – 3.69 (m, 2H), 2.68 – 2.26 (m, 4H). Step two: To a stirring solution of the above tosylate (0.50 g, 1.97 mmol) in DMF (10 mL) were added the commercially available 2-fluoro-5-nitrophenol (0.34 g, 2.16 mmol, 1.1 equiv.), potassium carbonate (0.41 g, 2.95 mmol, 1.5 equiv.) and potassium iodide (0.03 g, 0.20 mmol, 10 mol%). The reaction mixture was then stirred at 110 °C for 6 hours, after which the reaction was cooled to room temperature, diluted with ethyl acetate and washed with brine (3x). The organics were dried over $MgSO_4$ and concentrated *in vacuo*, after which the crude was purified by column chromatography eluting with 20-40% ethyl acetate in petroleum ether as eluent affording the title compound as an off-white solid. Yield: 50% (0.24 g, 0.98 mmol). ^{1}H NMR (400 MHz, $CDCl_3$) δ 7.99 – 7.83 (m, 2H), 7.22 (dd, J = 10.0, 8.8 Hz, 1H), 4.37 – 4.31 (m, 2H), 4.29 (d, J = 2.4 Hz, 2H), 4.02 – 3.93 (m, 2H), 2.51 (t, J = 2.4 Hz, 1H).

1-fluoro-4-nitro-2-(2-(2-(prop-2-yn-1-yloxy)ethoxy)ethoxy)benzene (**3c**). Step one: To a stirring solution of commercially available 2-(2-(prop-2-yn-1-yloxy)ethoxy)ethan-1-ol (1.0 g, 6.94 mmol) in dichloromethane (15 mL) were added triethylamine (1.93 mL, 13.9 mmol, 2.0 equiv.) and 4-dimethylaminopyridine (0.09 g, 0.69 mmol, 10 mol%). This solution was then cooled to 0 °C after which was added in dropwise manner tosyl chloride (1.32 g, 6.94 mmol, 1 equiv., in 20 mL dichloromethane). After completion of the addition the reaction mixture was allowed to warm to room temperature at which it was then maintained for 72 hours. Solvents were removed *in vacuo* and the crude product was purified by column chromatography eluting with 20-50% ethyl acetate in petroleum ether as eluent, affording the tosylate as a colourless oil. Yield: 71% (1.47 g, 4.90 mmol). ^{1}H NMR (400 MHz, $CDCl_3$) δ 7.79 (dd, J = 8.3, 2.5 Hz, 2H), 7.35 (d, J = 8.3 Hz, 2H), 4.23 – 4.11 (m, 4H), 3.72 – 3.66 (m, 2H), 3.65 – 3.57 (m, 4H), 2.47 (t, J = 2.5 Hz, 1H), 2.44 (s, 3H). Step two: To a stirring solution of the above tosylate (0.10 g, 0.34 mmol) in DMSO (5.0 mL) were added the commercially available 2-fluoro-5-nitrophenol (0.06 g, 0.37 mmol, 1.1 equiv.), potassium carbonate (0.07 g, 0.50 mmol, 1.5 equiv.) and potassium iodide (0.01 g, 0.03 mmol, 10 mol%). The reaction mixture was then stirred at 60 °C for 5 hours, after which the reaction was cooled to room temperature, diluted with ethyl acetate and washed with brine (3x). The organics were dried over $MgSO_4$ and concentrated *in vacuo*, after which the crude was purified by column chromatography eluting with 20-40% ethyl acetate in petroleum ether as eluent affording the title compound as an off-white solid. Yield: 59% (0.06 g, 0.20 mmol). ^{1}H NMR (400 MHz, $CDCl_3$) δ 7.93 (dd, J = 7.2, 2.7 Hz, 1H), 7.90 – 7.84 (m, 1H), 7.21 (dd, J = 10.0, 8.9 Hz, 1H), 4.34 – 4.29 (m, 2H), 4.21 (d, J = 2.4 Hz, 2H), 3.98 – 3.92 (m, 2H), 3.82 – 3.70 (m, 4H), 2.45 (t, J = 2.4 Hz, 1H).

*4-Chloro-N-(5-chloro-2-(4-nitro-2-(prop-2-yn-1-yloxy)phenoxy)phenyl)-3-(trifluoromethyl)benzenesulfonamide (**4a**).* To a stirring solution of 4-chloro-*N*-(5-chloro-2-hydroxyphenyl)-3-(trifluoromethyl)benzenesulfonamide (0.43 g, 1.1 mmol) and 1-fluoro-4-nitro-2-(prop-2-yn-1-yloxy)benzene (0.23 g, 1.1 mmol) in DMF (10 mL) was added K_2CO_3 (0.38 g, 2.75 mmol, 2.5 equiv.) and mixture was left to stir at 70 °C for 16 hours. The reaction mixture was then cooled to room temperature, diluted with ethyl acetate and washed subsequently with 1N HCl (1x), H_2O (1x) and brine (1x). The organics were dried over $MgSO_4$, concentrated *in vacuo* and purified by column chromatography eluting with 10-20% ethyl acetate in petroleum ether, affording the title compound as a yellow solid. Yield: 58% (0.36 g, 0.64 mmol). ^{1}H NMR (400 MHz, $CDCl_3$) δ 8.09 (s, 1H), 7.99 (d, J = 2.4 Hz, 1H), 7.88 (dd, J = 8.4, 1.7 Hz, 1H), 7.80 (dd, J = 8.9, 2.4 Hz, 1H), 7.72 (d, J = 2.3 Hz, 1H), 7.57 (d, J = 8.4 Hz, 1H), 7.33 (s, 1H), 7.08 (dd, J = 8.8, 2.4 Hz, 1H), 6.71 – 6.63 (m, 2H), 4.77 (d, J = 2.2 Hz, 2H), 2.64 (t, J = 2.1 Hz, 1H).

*4-chloro-N-(5-chloro-2-(4-nitro-2-(2-(prop-2-yn-1-yloxy)ethoxy)phenoxy)phenyl)-3-(trifluoromethyl)benzenesulfonamide (**4b**)*. To a stirring solution of 1-fluoro-4-nitro-2-(2-(prop-2-yn-1-yloxy)ethoxy)benzene (0.33 g, 1.37 mmol) in DMSO (4.0 mL) were added 4-chloro-*N*-(5-chloro-2-hydroxyphenyl)-3-(trifluoromethyl)benzenesulfonamide (0.42 g, 1.10 mmol, 0.8 equiv.) and potassium carbonate (0.38 g, 2.74 mmol, 2.0 equiv.). This solution was stirred at 60 °C for 16 hours, after which TLC indicated full consumption of phenol intermediate. The reaction mixture was cooled to room temperature, diluted with ethyl acetate and washed with brine (3x). The organics were dried over $MgSO_4$, concentrated *in vacuo*, and the crude product was purified by column chromatography eluting with 0-20% ethyl acetate in petroleum ether as eluent. A final recrystallization from a 1:1 mixture of diethyl ether and hexanes afforded the title compound as a white solid. Yield: 56% (0.47 g, 0.77 mmol). ^{1}H NMR (400 MHz, $CDCl_3$) *δ* 8.09 (d, *J* = 2.2 Hz, 1H), 7.88 (dd, *J* = 8.4, 2.2 Hz, 1H), 7.85 – 7.80 (m, 1H), 7.77 (dd, *J* = 8.8, 2.5 Hz, 1H), 7.68 (d, *J* = 2.5 Hz, 1H), 7.62 (s, 1H), 7.56 (d, *J* = 8.4 Hz, 1H), 7.08 (dd, *J* = 8.7, 2.5 Hz, 1H), 6.78 (d, *J* = 8.8 Hz, 1H), 6.70 (d, *J* = 8.7 Hz, 1H), 4.27 – 4.20 (m, 4H), 3.87 – 3.78 (m, 2H), 2.49 (t, *J* = 2.4 Hz, 1H).

*4-chloro-N-(5-chloro-2-(4-nitro-2-(2-(2-(prop-2-yn-1-yloxy)ethoxy)ethoxy)phenoxy)phenyl)-3-(trifluoromethyl)benzenesulfonamide (**4c**).* To a stirring solution of 1-fluoro-4-nitro-2-(2-(2-(prop-2-yn-1-yloxy)ethoxy)ethoxy)benzene (0.34 g, 1.20 mmol) in DMSO (4.0 mL) were added 4-chloro-*N*-(5-chloro-2-hydroxyphenyl)-3-(trifluoromethyl)benzenesulfonamide (0.37 g, 0.96 mmol, 0.8 equiv.) and potassium carbonate (0.33 g, 2.40 mmol, 2.0 equiv.). This solution was stirred at 60 °C for 72 hours, after which the reaction mixture was cooled to room temperature, diluted with ethyl acetate and washed with brine (3x). The organics were dried over $MgSO_4$, concentrated *in vacuo*, and the crude was purified by column chromatography eluting with 10-30% ethyl acetate in petroleum ether as eluent, affording the title compound as a light brown solid. Yield: 51% (0.40 g, 0.62 mmol). ^{1}H NMR (400 MHz, $CDCl_3$) *δ* 8.17 – 8.04 (m, 2H), 7.88 (dd, *J* = 8.4, 2.3 Hz, 1H), 7.79 (d, *J* = 2.6 Hz, 1H), 7.70 (dd, *J* = 8.8, 2.6 Hz, 1H), 7.63 (d, *J* = 2.5 Hz, 1H), 7.53 (d, *J* = 8.4 Hz, 1H), 7.06 (dd, *J* = 8.8, 2.5 Hz, 1H), 6.69 (d, *J* = 3.6 Hz, 1H), 6.67 (d, *J* = 3.6 Hz, 1H), 4.26 – 4.14 (m, 4H), 3.83 – 3.72 (m, 2H), 3.72 – 3.63 (m, 4H), 2.43 (t, *J* = 2.4 Hz, 1H).

*N-(2-(4-amino-2-(prop-2-yn-1-yloxy)phenoxy)-5-chlorophenyl)-4-chloro-3-(trifluoromethyl)benzenesulfonamide (**5a**).* To a stirring solution of 4-chloro-*N*-(5-chloro-2-(4-nitro-2-(prop-2-yn-1-yloxy)phenoxy)phenyl)-3-(trifluoromethyl)benzenesulfonamide (1.40 g, 2.5 mmol) in ethyl acetate (50 mL) was added tin (II) chloride dihydrate (2.80 g, 12.6 mmol, 5.0 equiv.) and the resulted solution was stirred at 40 °C for 20 hours. The reaction mixture was left to cool to room temperature, quenched by addition of sat. $NaHCO_3$ (25 mL) and subsequently extracted with ethyl acetate (3x). The combined organics were washed with brine, dried over $MgSO_4$ and concentrated *in vacuo*. The crude was purified by column chromatography eluting with 1% MeOH in dichloromethane, affording the title compound as a brown solid. Yield: 71% (0.94 g, 1.78 mmol). ^{1}H NMR (400 MHz, $CDCl_3$) *δ* 8.13 (s, 1H), 7.88 (dd, *J* = 8.4, 2.2 Hz, 1H), 7.62 (d, *J* = 2.5 Hz, 1H), 7.58 (d, *J* = 8.4 Hz, 1H), 6.94 (dd, *J* = 8.8, 2.5 Hz, 1H), 6.51 (d, *J* = 8.8 Hz, 1H), 6.45 (d, *J* = 8.5 Hz, 1H), 6.42 (d, *J* = 2.5 Hz, 1H), 6.23 (dd, *J* = 8.5, 2.5 Hz, 1H), 4.51 (d, *J* = 2.4 Hz, 2H), 2.51 (t, *J* = 2.4 Hz, 1H).

*N-(2-(4-amino-2-(2-(prop-2-yn-1-yloxy)ethoxy)phenoxy)-5-chlorophenyl)-4-chloro-3-(trifluoromethyl)benzenesulfonamide (**5b**).* To a roundbottom flask containing 4-chloro-*N*-(5-chloro-2-(4-nitro-2-(2-(prop-2-yn-1-yloxy)ethoxy)phenoxy)phenyl)-3-(trifluoromethyl)benzenesulfonamide (0.47 g, 0.77 mmol) in ethyl acetate (10 mL) was added tin (II) chloride dihydrate (0.87 g, 3.86 mmol, 5.0 equiv.) and the solution was subsequently stirred at 40 °C for 16 hours. TLC indicated full conversion to the desired aniline so the reaction mixture was cooled to room temperature and quenched by addition of aqueous NaOH (10 mL, 3.0M). Layers were separated and the aqueous was extracted with

ethyl acetate (3x). The combined organics were washed with brine, dried over $MgSO_4$ and concentrated *in vacuo*. The crude was purified by column chromatography eluting with dichloromethane as eluent and affording the title compound as a brown solid. Yield: 50% (0.22 g, 0.39 mmol). ^{1}H NMR (400 MHz, $CDCl_3$) δ 8.10 (d, *J* = 2.2 Hz, 1H), 7.83 (dd, *J* = 8.4, 2.3 Hz, 1H), 7.59 (d, *J* = 2.5 Hz, 1H), 7.54 (d, *J* = 8.4 Hz, 1H), 6.95 (dd, *J* = 8.8, 2.5 Hz, 1H), 6.56 (d, *J* = 8.8 Hz, 1H), 6.49 (d, *J* = 8.4 Hz, 1H), 6.25 (d, *J* = 2.5 Hz, 1H), 6.19 (dd, *J* = 8.4, 2.5 Hz, 1H), 4.14 (d, *J* = 2.4 Hz, 2H), 4.01 – 3.92 (m, 2H), 3.71 – 3.64 (m, 2H), 2.45 (t, *J* = 2.4 Hz, 1H).

*N-(2-(4-amino-2-(2-(2-(prop-2-yn-1-yloxy)ethoxy)ethoxy)phenoxy)-5-chlorophenyl)-4-chloro-3-(trifluoromethyl)benzenesulfonamide (**5c**).* To a roundbottom flask containing 4-chloro-*N*-(5-chloro-2-(4-nitro-2-(2-(2-(prop-2-yn-1-yloxy)ethoxy)ethoxy)phenoxy)phenyl)-3-(trifluoromethyl)benzenesulfonamide (0.07 g, 0.11 mmol) in ethyl acetate (5.0 mL) was added tin (II) chloride dihydrate (0.19 g, 0.83 mmol, 7.5 equiv.) and the solution was subsequently stirred at 40 °C for 32 hours. TLC indicated full conversion to the desired aniline so the reaction mixture was cooled to room temperature and quenched by addition of aqueous NaOH (5.0 mL, 3.0M). Layers were separated and the aqueous was extracted with ethyl acetate (3x). The combined organics were washed with brine, dried over $MgSO_4$ and concentrated *in vacuo*. The crude was purified by column chromatography eluting with 0-5% MeOH in dichloromethane as eluent and affording the title compound as a light brown solid. Yield: 38% (0.03 g, 0.04 mmol). ^{1}H NMR (400 MHz, $CDCl_3$) δ 8.11 (d, *J* = 2.2 Hz, 1H), 7.85 (dd, *J* = 8.4, 2.3 Hz, 1H), 7.60 (d, *J* = 2.5 Hz, 1H), 7.56 (d, *J* = 8.5 Hz, 1H), 6.96 (dd, *J* = 8.8, 2.5 Hz, 1H), 6.56 (d, *J* = 8.8 Hz, 1H), 6.50 (d, *J* = 8.5 Hz, 1H), 6.25 (d, *J* = 2.5 Hz, 1H), 6.18 (dd, *J* = 8.5, 2.5 Hz, 1H), 4.19 (d, *J* = 2.4 Hz, 2H), 4.00 – 3.93 (m, 2H), 3.69 – 3.63 (m, 4H), 3.60 (ddd, *J* = 7.0, 3.2, 1.4 Hz, 2H), 2.44 (t, *J* = 2.4 Hz, 1H).

*N-(4-(4-chloro-2-((4-chloro-3-(trifluoromethyl)phenyl)sulfonamido)phenoxy)-3-(prop-2-yn-1-yloxy)phenyl)acrylamide (**6a**).* To a stirring solution of *N*-(2-(4-amino-2-(prop-2-yn-1-yloxy)phenoxy)-5-chlorophenyl)-4-chloro-3-(trifluoromethyl)benzenesulfonamide (0.29 g, 0.54 mmol) in ethyl acetate (10 mL) were added acryloyl chloride (0.1 g, 1.1 mmol, 2.1 equiv.), $NaHCO_3$ (0.08 g, 0.94 mmol, 1.75 equiv.), and H_2O (10 mL). The resulted suspension was stirred for 20 minutes, after which TLC showed full consumption of the aniline. The reaction mixture was diluted with H_2O, extracted with ethyl acetate (3x), the combined organics washed with brine and dried over $MgSO_4$. The crude product was purified by column chromatography, eluting with 1% MeoH in DCM to give the title compound as a brown solid. Yield: 17% (29 mg, 0.05 mmol). ^{1}H NMR (400 MHz, $CDCl_3$) δ 8.11 (d, *J* = 2.2 Hz, 1H), 7.88 (dd, *J* = 8.4, 2.3 Hz, 1H), 7.73 (s, 1H), 7.65 (d, *J* = 2.5 Hz, 1H), 7.58 (d, *J* = 8.4 Hz, 1H), 7.43 (s, 1H), 7.40 (s, 1H), 6.97 (dd, *J* = 8.8, 2.5 Hz, 1H), 6.91 (dd, *J* = 8.7, 2.4 Hz, 1H), 6.60 (d, *J* = 8.6 Hz, 1H), 6.54 (d, *J* = 8.8 Hz, 1H), 6.50 – 6.42 (m, 1H), 6.31 – 6.20 (m, 1H), 5.82 (dd, *J* = 10.1, 1.2 Hz, 1H), 4.61 (d, *J* = 2.4 Hz, 2H), 2.54 (t, *J* = 2.6 Hz, 1H). LC−MS (ESI) *m/z* calcd for $C_{25}H_{17}Cl_2F_3N_2O_5S$ $[M + H]^+$ 585.03, found 585.00, 586.95. HPLC: 11.9 min, purity 95%.

*N-(4-(4-chloro-2-((4-chloro-3-(trifluoromethyl)phenyl)sulfonamido)phenoxy)-3-(2-(prop-2-yn-1-yloxy)ethoxy)phenyl)acrylamide (**6b**).* A stirring solution of *N*-(2-(4-amino-2-(2-(prop-2-yn-1-yloxy)ethoxy)phenoxy)-5-chlorophenyl)-4-chloro-3-(trifluoromethyl)benzenesulfonamide (0.06 g, 0.11 mmol) in dichloromethane (20 mL) was cooled to ~-10 °C with a salt/ice bath after which was added acryloyl chloride (8.90 µL, 1.1 mmol, 1.0 equiv.). After one hour of stirring the salt/ice bath was removed and the reaction mixture was left to stir at room temperature for an additional hour after which TLC indicated full conversion. The solvents were removed *in vacuo* and the crude was purified by semi-preparative HPLC affording the title compound as an off-white solid. Yield: 35% (24 mg, 0.04 mmol). ^{1}H NMR (400 MHz, $CDCl_3$) δ 8.10 (d, *J* = 2.2 Hz, 1H), 7.86 (dd, *J* = 8.4, 2.3 Hz, 1H), 7.69 – 7.61 (m, 3H), 7.55 (d, *J* = 8.4 Hz, 1H), 7.27 (s, 1H), 7.00 (dd, *J* = 8.8, 2.5 Hz, 1H), 6.80 (dd, *J* = 8.6, 2.4 Hz, 1H), 6.68

(d, *J* = 8.6 Hz, 1H), 6.61 (d, *J* = 8.8 Hz, 1H), 6.45 (dd, *J* = 16.8, 1.2 Hz, 1H), 6.23 (dd, *J* = 16.4, 10.0 Hz, 1H), 5.81 (dd, *J* = 10.2, 1.2 Hz, 1H), 4.19 (d, *J* = 2.4 Hz, 2H), 4.15 – 4.07 (m, 2H), 3.77 – 3.70 (m, 2H), 2.45 (t, *J* = 2.4 Hz, 1H). LC–MS (ESI) *m/z* calcd for $C_{27}H_{21}Cl_2F_3N_2O_6S$ $[M + H]^+$ 629.05, found 629.15, 631.15. HPLC: 12.0 min, purity 99%.

*N-(4-(4-chloro-2-((4-chloro-3-(trifluoromethyl)phenyl)sulfonamido)phenoxy)-3-(2-(2-(prop-2-yn-1-yloxy)ethoxy)ethoxy)phenyl)acrylamide (**6c**).* To a stirring solution of *N*-(2-(4-amino-2-(2-(2-(prop-2-yn-1-yloxy)ethoxy)ethoxy)phenoxy)-5-chlorophenyl)-4-chloro-3-(trifluoromethyl)benzenesulfonamide (0.08 g, 0.13 mmol) in dichloromethane (20 mL) was cooled to ~-10 °C with a salt/ice bath after which was added acryloyl chloride (27.0 µL, 0.33 mmol, 2.6 equiv.). After one hour of stirring the salt/ice bath was removed and the reaction mixture was left to stir at room temperature for an additional hour after which TLC indicated full conversion. The solvents were removed *in vacuo* and the crude was purified by semi-preparative HPLC affording the title compound as an off-white solid. Yield: 37% (31 mg, 0.05 mmol). ^{1}H NMR (400 MHz, $CDCl_3$) *δ* 8.11 (s, 1H), 8.04 (s, 1H), 7.88 (dd, *J* = 8.4, 2.2 Hz, 1H), 7.66 – 7.60 (m, 2H), 7.59 (s, 1H), 7.54 (d, *J* = 8.4 Hz, 1H), 6.99 (dd, *J* = 8.8, 2.5 Hz, 1H), 6.83 (dd, *J* = 8.6, 2.4 Hz, 1H), 6.58 (dd, *J* = 8.7, 7.2 Hz, 2H), 6.50 – 6.40 (m, 1H), 6.33 – 6.20 (m, 1H), 5.79 (d, *J* = 10.1, 1.4 Hz, 1H), 4.19 (d, *J* = 2.4 Hz, 2H), 4.11 – 4.01 (m, 2H), 3.74 – 3.66 (m, 4H), 3.66 – 3.61 (m, 2H), 2.44 (t, *J* = 2.3 Hz, 1H), 2.20 (d, *J* = 18.1 Hz, 1H). LC–MS (ESI) *m/z* calcd for $C_{29}H_{25}Cl_2F_3N_2O_7S$ $[M + H]^+$ 673.08, found 673.15, 675.15. HPLC: 12.0 min, purity 96%.

*3-bromo-N-(4-(4-chloro-2-((4-chloro-3-(trifluoromethyl)phenyl)sulfonamido)phenoxy)-3-(prop-2-yn-1-yloxy)phenyl)propanamide (**7a**).* To a stirring solution of *N*-(2-(4-amino-2-(prop-2-yn-1-yloxy)phenoxy)-5-chlorophenyl)-4-chloro-3-(trifluoromethyl)benzenesulfonamide (0.32 g, 0.60 mmol) in ethyl acetate (10 mL) were added 3-bromopropanoyl chloride (0.21 g, 1.13 mmol, 2.1 equiv.), $NaHCO_3$ (0.09 g, 1.04 mmol, 1.75 equiv.), and H_2O (10 mL). The resulted suspension was stirred for 20 minutes, after which TLC showed full consumption of the aniline. The reaction mixture was diluted with H_2O, extracted with ethyl acetate (3x), the combined organics washed with brine and dried over $MgSO_4$. The crude product was purified by column chromatography, eluting with 2% MeoH in DCM to give the title compound as a white solid. Yield: 82% (0.46 g, 0.69 mmol). ^{1}H NMR (400 MHz, $CDCl_3$) *δ* 8.11 (d, *J* = 2.0 Hz, 1H), 7.88 (dd, *J* = 8.4, 2.1 Hz, 1H), 7.65 (d, *J* = 2.5 Hz, 1H), 7.61 (d, *J* = 2.3 Hz, 1H), 7.58 (d, *J* = 8.4 Hz, 1H), 7.41 (s, 1H), 7.36 (s, 1H), 6.97 (dd, *J* = 8.8, 2.5 Hz, 1H), 6.88 (dd, *J* = 8.6, 2.3 Hz, 1H), 6.60 (d, *J* = 8.6 Hz, 1H), 6.54 (d, *J* = 8.8 Hz, 1H), 4.61 (d, *J* = 2.3 Hz, 2H), 3.72 (t, *J* = 6.4 Hz, 2H), 2.97 (t, *J* = 6.4 Hz, 2H), 2.54 (t, *J* = 2.3 Hz, 1H).

*3-bromo-N-(4-(4-chloro-2-((4-chloro-3-(trifluoromethyl)phenyl)sulfonamido)phenoxy)-3-(2-(prop-2-yn-1-yloxy)ethoxy)phenyl)propanamide (**7b**).* To a stirring solution of *N*-(2-(4-amino-2-(2-(prop-2-yn-1-yloxy)ethoxy)phenoxy)-5-chlorophenyl)-4-chloro-3-(trifluoromethyl)benzenesulfonamide (58 mg, 0.10 mmol) in ethyl acetate (2.0 mL) were added 3-bromopropanoyl chloride (36 mg, 0.21 mmol, 2.1 equiv.), and H_2O (2.0 mL). The resulted suspension was stirred for 30 minutes, after which TLC showed full consumption of the aniline. The reaction mixture was diluted with ethyl acetate and H_2O, layers separated, and the aqueous extracted with ethyl acetate (2x). The combined organics were washed with brine (2x), dried over $MgSO_4$ and concentrated *in vacuo* after which the crude product was taken up in acetonitrile and washed with hexanes (2x) to afford the title compound as an off-white solid. Yield: 95% (68 mg, 0.10 mmol). ^{1}H NMR (400 MHz, $CDCl_3$) *δ* 8.11 (d, *J* = 2.2 Hz, 1H), 7.87 (dd, *J* = 8.4, 2.3 Hz, 1H), 7.76 (br s, 1H), 7.64 (br s, 1H), 7.61 (d, *J* = 2.5 Hz, 1H), 7.55 (d, *J* = 8.4 Hz, 1H), 7.51 (d, *J* = 2.4 Hz, 1H), 6.99 (dd, *J* = 8.8, 2.5 Hz, 1H), 6.81 (dd, *J* = 8.6, 2.4 Hz, 1H), 6.65 (d, *J* = 8.6 Hz, 1H), 6.58 (d, *J* = 8.8 Hz, 1H), 4.18 (d, *J* = 2.4 Hz, 2H), 4.12 – 4.02 (m, 2H), 3.77 – 3.64 (m, 4H), 3.00 – 2.92 (m, 2H), 2.46 (t, *J* = 2.4 Hz, 1H).

*3-bromo-N-(4-(4-chloro-2-((4-chloro-3-(trifluoromethyl)phenyl)sulfonamido)phenoxy)-3-(2-(2-(prop-2-yn-1-yloxy)ethoxy)ethoxy)phenyl)propanamide (**7c**).* A small flask was charged with *N*-(2-(4-amino-2-(2-(2-(prop-2-yn-1-yloxy)ethoxy)ethoxy)phenoxy)-5-chlorophenyl)-4-chloro-3-(trifluoromethyl)benzenesulfonamide (77 mg, 0.12 mmol), ethyl acetate (3.0 mL) and H_2O (3.0 mL). To this solution was then added 3-bromopropanoyl chloride (45 mg, 0.26 mmol, 2.1 equiv.) and the reaction was left to stir for 5 hours. The reaction mixture then was diluted with ethyl acetate and H_2O, layers separated, and the aqueous extracted with ethyl acetate (2x). The combined organics were washed with brine (2x), dried over $MgSO_4$ and concentrated *in vacuo* after which the crude product was taken up in acetonitrile and washed with hexanes (2x) to afford the title compound as an off-white solid. Yield: 77% (72 mg, 0.10 mmol). ^{1}H NMR (300 MHz, $CDCl_3$) δ 8.11 (d, *J* = 2.3 Hz, 1H), 8.00 – 7.83 (m, 2H), 7.72 – 7.63 (m, 1H), 7.62 – 7.59 (m, 1H), 7.55 (d, *J* = 8.4 Hz, 1H), 7.47 (d, *J* = 2.4 Hz, 1H), 6.98 (dd, *J* = 8.8, 2.5 Hz, 1H), 6.82 (dd, *J* = 8.6, 2.3 Hz, 1H), 6.67 – 6.50 (m, 2H), 4.19 (d, *J* = 2.4 Hz, 2H), 4.09 – 3.97 (m, 2H), 3.75 – 3.60 (m, 8H), 2.95 (t, *J* = 6.5 Hz, 2H), 2.45 (t, *J* = 2.2 Hz, 1H).

*N-(4-(4-chloro-2-((4-chloro-3-(trifluoromethyl)phenyl)sulfonamido)phenoxy)-3-(prop-2-yn-1-yloxy)phenyl)-3-thiocyanatopropanamide (**8a**).* To a roundbottom flask containing ethanol (35 mL) were added 3-bromo-*N*-(4-(4-chloro-2-((4-chloro-3-(trifluoromethyl)phenyl)sulfonamido)phenoxy)-3-(prop-2-yn-1-yloxy)phenyl)propanamide (0.19 g, 0.28 mmol) and potassium thiocyanate (0.16 g, 1.70 mmol, 6.0 equiv.). The solution was then heated to reflux and stirred for 48 hours, after which the reaction mixture was concentrated *in vacuo*¸ taken up in ethyl acetate and washed with H_2O and brine, respectively. The organics were dried over $MgSO_4$, concentrated *in vacuo* and the resulted crude product was purified by column chromatography eluting with 2% MeOH in dichloromethane to yield the title compound as a white solid. Yield: 53% (0.10 g, 0.16 mmol). ^{1}H NMR (400 MHz, $CDCl_3$) δ 8.12 (d, *J* = 1.9 Hz, 1H), 7.89 (dd, *J* = 8.4, 2.1 Hz, 1H), 7.77 (s, 1H), 7.64 (d, *J* = 2.5 Hz, 1H), 7.61 – 7.54 (m, 2H), 7.51 (s, 1H), 7.01 – 6.89 (m, 2H), 6.59 (d, *J* = 8.6 Hz, 1H), 6.54 (d, *J* = 8.8 Hz, 1H), 4.61 (d, *J* = 2.3 Hz, 2H), 3.33 (t, *J* = 6.5 Hz, 2H), 2.95 (t, *J* = 6.5 Hz, 2H), 2.55 (t, *J* = 2.3 Hz, 1H). LC–MS (ESI) *m/z* calcd for $C_{26}H_{18}Cl_2F_3N_3O_5S_2$ $[M + H]^+$ 644.01, found 643.95, 645.95. HPLC: 12.1 min, purity 99%.

*N-(4-(4-chloro-2-((4-chloro-3-(trifluoromethyl)phenyl)sulfonamido)phenoxy)-3-(2-(prop-2-yn-1-yloxy)ethoxy)phenyl)-3-thiocyanatopropanamide (**8b**).* To a roundbottom flask containing ethanol (10 mL) were added 3-bromo-*N*-(4-(4-chloro-2-((4-chloro-3-(trifluoromethyl)phenyl)sulfonamido)phenoxy)-3-(2-(prop-2-yn-1-yloxy)ethoxy)phenyl)propanamide (65 mg, 0.09 mmol) and potassium thiocyanate (88 mg, 0.91 mmol, 10.0 equiv.). The solution was then heated to reflux and stirred for 30 hours, after which the reaction mixture was concentrated *in vacuo*¸ taken up in ethyl acetate and washed with H_2O and brine, respectively. The organics were dried over $MgSO_4$, concentrated *in vacuo* and the resulted crude product was purified by column chromatography eluting with 1% MeOH in dichloromethane to yield the title compound as a brown solid. Yield: 82% (51 mg, 0.08 mmol). ^{1}H NMR (400 MHz, $CDCl_3$) δ 8.11 (d, *J* = 2.2 Hz, 1H), 7.87 (dd, *J* = 8.4, 2.3 Hz, 1H), 7.83 (br s, 1H), 7.69 (br s, 1H), 7.61 (d, *J* = 2.5 Hz, 1H), 7.56 (d, *J* = 8.4 Hz, 1H), 7.47 (d, *J* = 2.4 Hz, 1H), 6.99 (dd, *J* = 8.8, 2.5 Hz, 1H), 6.85 (dd, *J* = 8.6, 2.4 Hz, 1H), 6.67 (d, *J* = 8.6 Hz, 1H), 6.58 (d, *J* = 8.7 Hz, 1H), 4.18 (d, *J* = 2.4 Hz, 2H), 4.11 – 4.05 (m, 2H), 3.76 – 3.69 (m, 2H), 3.31 (t, *J* = 6.5 Hz, 2H), 2.93 (t, *J* = 6.5 Hz, 2H), 2.46 (t, *J* = 2.4 Hz, 1H). LC–MS (ESI) *m/z* calcd for $C_{28}H_{22}Cl_2F_3N_3O_6S_2$ $[M + H]^+$ 688.04, found 688.20, 690.20. HPLC: 12.1 min, purity 97%.

*N-(4-(4-chloro-2-((4-chloro-3-(trifluoromethyl)phenyl)sulfonamido)phenoxy)-3-(2-(2-(prop-2-yn-1-yloxy)ethoxy)ethoxy)phenyl)-3-thiocyanatopropanamide (**8c**).* To a roundbottom flask containing ethanol (10 mL) were added 3-bromo-*N*-(4-(4-chloro-2-((4-chloro-3-(trifluoromethyl)phenyl)sulfonamido)phenoxy)-3-(2-(2-(prop-2-yn-1-yloxy)ethoxy)ethoxy)phenyl)propanamide (72 mg, 0.10 mmol) and potassium thiocyanate (92 mg, 0.95

mmol, 10.0 equiv.). The solution was then heated to reflux and stirred for 23 hours, after which the reaction mixture was concentrated *in vacuo*¸ taken up in ethyl acetate and washed with H_2O and brine, respectively. The organics were dried over $MgSO_4$, concentrated *in vacuo* and the resulted crude product was purified by column chromatography eluting with 1% MeOH in dichloromethane to yield the title compound as a brown solid. Yield: 66% (46 mg, 0.06 mmol). ^{1}H NMR (400 MHz, $CDCl_3$) δ 8.15 – 8.10 (m, 1H), 7.98 – 7.81 (m, 3H), 7.66 – 7.59 (m, 1H), 7.56 (d, J = 8.4 Hz, 1H), 7.46 – 7.41 (m, 1H), 6.98 (d, J = 8.8 Hz, 1H), 6.85 (d, J = 8.6 Hz, 1H), 6.61 (d, J = 8.6 Hz, 1H), 6.57 (d, J = 8.8 Hz, 1H), 4.24 – 4.16 (m, 2H), 4.09 – 4.00 (m, 2H), 3.72 – 3.66 (m, 5H), 3.66 – 3.61 (m, 2H), 3.30 (t, J = 6.4 Hz, 2H), 2.93 (t, J = 6.4 Hz, 2H), 2.51 – 2.39 (m, 1H). LC–MS (ESI) *m/z* calcd for $C_{30}H_{26}Cl_2F_3N_3O_7S_2$ $[M + H]^+$ 732.06, found 732.15, 734.15. HPLC: 12.1 min, purity 99%.

*4-chloro-N-(5-chloro-2-(4-nitrophenoxy)phenyl)-3-(trifluoromethyl)benzenesulfonamide (**9**).* To a roundbottom flask were added commercially available 1-fluoro-4-nitrobenzene (73 mg, 0.52 mmol), 4-chloro-N-(5-chloro-2-hydroxyphenyl)-3-(trifluoromethyl)benzenesulfonamide (0.20 g, 0.52 mmol, 1.0 equiv.), potassium carbonate (0.14 g, 1.04 mmol, 2.0 equiv.), and DMSO (3.0 mL). This solution was then left to stir at 60 °C for 20 hours, after which the reaction mixture was cooled to room temperature, diluted with ethyl acetate and washed with brine (3x). The organics were dried over $MgSO_4$, concentrated *in vacuo* and the resulting crude purified by column chromatography, eluting with 10-30% ethyl acetate in petroleum ether as eluent and affording the title compound as a white solid. Yield: 56% (0.15 g, 0.29 mmol). ^{1}H NMR (400 MHz, $CDCl_3$) δ 8.17 – 8.11 (m, 2H), 8.04 (d, J = 2.3 Hz, 1H), 7.83 (dd, J = 8.4, 2.3 Hz, 1H), 7.75 (d, J = 2.4 Hz, 1H), 7.54 (d, J = 8.4 Hz, 1H), 7.16 (dd, J = 8.7, 2.5 Hz, 1H), 7.06 (br s, 1H), 6.83 (d, J = 8.7 Hz, 1H), 6.79 – 6.70 (m, 2H).

*N-(2-(4-aminophenoxy)-5-chlorophenyl)-4-chloro-3-(trifluoromethyl)benzenesulfonamide (**10**).* To a stirring solution containing 4-chloro-*N*-(5-chloro-2-(4-nitrophenoxy)phenyl)-3-(trifluoromethyl)benzenesulfonamide (0.15 g, 0.29 mmol) in ethyl acetate (25 mL) was added tin(II) chloride dihydrate (1.31 g, 5.80 mmol, 20.0 equiv.) and was left to stir at 40 °C for 72 hours. The reaction mixture was cooled to room temperature, quenched by addition of aqueous NaOH (25.0 mL, 2.0M). Layers were separated and the aqueous was extracted with ethyl acetate (3x). The combined organics were washed with brine, dried over $MgSO_4$ and concentrated *in vacuo*. The crude was purified by column chromatography eluting with 0-1% MeOH in dichloromethane as eluent and affording the title compound as an off-white solid. Yield: 99% (0.14 g, 0.29 mmol). ^{1}H NMR (400 MHz, $CDCl_3$) δ 8.09 (d, J = 2.3 Hz, 1H), 7.84 (dd, J = 8.4, 2.3 Hz, 1H), 7.64 (d, J = 2.5 Hz, 1H), 7.57 (d, J = 8.4 Hz, 1H), 6.98 (dd, J = 8.8, 2.5 Hz, 1H), 6.64 – 6.56 (m, 2H), 6.54 (d, J = 8.8 Hz, 1H), 6.45 – 6.36 (m, 2H), 3.65 (br s, 2H).

*N-(4-(4-chloro-2-((4-chloro-3-(trifluoromethyl)phenyl)sulfonamido)phenoxy)phenyl)propionamide (**11**).* Step one: To a stirring solution of *N*-(2-(4-aminophenoxy)-5-chlorophenyl)-4-chloro-3-(trifluoromethyl)benzenesulfonamide (0.11 g, 0.23 mmol) in dichloromethane (20 mL) was added in dropwise fashion acryloyl chloride (45 µL, 0.55 mmol, 2.4 equiv., in 20 mL dichloromethane). After completion of the addition the reaction mixture was left to stir for 18 hours after which the solvents were removed *in vacuo* and the crude purified by column chromatography eluting with 0-1% MeOH in dichloromethane. Yield: 25% (30 mg, 0.06 mmol, brown solid). ^{1}H NMR (400 MHz, $CDCl_3$) δ 8.08 (d, J = 2.3 Hz, 1H), 7.83 (dd, J = 8.4, 2.3 Hz, 1H), 7.67 (d, J = 2.5 Hz, 1H), 7.55 (d, J = 8.4 Hz, 1H), 7.52 – 7.45 (m, 3H), 7.26 – 7.19 (m, 1H), 7.04 (dd, J = 8.8, 2.5 Hz, 1H), 6.63 (d, J = 8.8 Hz, 1H), 6.61 – 6.54 (m, 2H), 6.45 (dd, J = 16.8, 1.3 Hz, 1H), 6.26 (dd, J = 16.8, 10.2 Hz, 1H), 5.80 (dd, J = 10.2, 1.3 Hz, 1H). Step two: A small roundbottom flask was charged with the above acrylamide (53 mg, 0.10 mmol) and MeOH (5.0 mL) and the flask was purged with N_2 three times. A spatula tip of Pd/C (10% wt) was added and the flask was again purged with N_2 three times. The reaction was then put under a H_2 atmosphere and the reaction was left to stir for 4 hours. Upon completion, the reaction mixture was filtered over Celite,

solvents removed *in vacuo* and the crude purified by column chromatography eluting with dichloromethane. A final recrystallization from chloroform/pentane afforded the title compound as a white crystalline solid. Yield: 75% (40 mg, 0.08 mmol). ^{1}H NMR (400 MHz, $CDCl_3$) δ 8.07 (d, J = 2.2 Hz, 1H), 7.82 (dd, J = 8.4, 2.3 Hz, 1H), 7.68 (d, J = 2.5 Hz, 1H), 7.55 (d, J = 8.5 Hz, 1H), 7.47 – 7.35 (m, 2H), 7.23 – 7.13 (m, 2H), 7.03 (dd, J = 8.8, 2.5 Hz, 1H), 6.62 (d, J = 8.8 Hz, 1H), 6.60 – 6.51 (m, 2H), 2.40 (q, J = 7.6 Hz, 2H), 1.26 (t, J = 7.5 Hz, 3H). LC–MS (ESI) *m/z* calcd for $C_{22}H_{17}Cl_2F_3N_2O_4S$ $[M + H]^+$ 533.03, found 533.05, 535.05. HPLC: 11.8 min, purity 95%.

*N-(2-(4-amino-2-propoxyphenoxy)-5-chlorophenyl)-4-chloro-3-(trifluoromethyl)benzenesulfonamide (**12**).* To a stirring solution of **5a** (115 mg, 0.22 mmol) in MeOH (5 mL) was added a catalytic amount of Pd/C (10% wt) and the flask was purged with N_2 three times. The reaction was then put under a H_2 atmosphere and left to stir for 3 hours at room temperature. Upon completion, the reaction mixture was filtered over Celite and concentrated in vacuo. The crude product was purified by column chromatography eluting with 0-1% MeOH in DCM. Yield: quant. (116 mg, 0.22 mmol). ^{1}H NMR: (400 MHz, $CDCl_3$) δ 8.11 (d, J = 2.3 Hz, 1H), 7.83 (dd, J = 8.4, 2.3 Hz, 1H), 7.61 (d, J = 2.5 Hz, 1H), 7.55 (d, J = 8.4 Hz, 1H), 6.93 (dd, J = 8.8, 2.5 Hz, 1H), 6.54 (d, J = 8.8 Hz, 1H), 6.50 (d, J = 8.4 Hz, 1H), 6.24 (d, J = 2.5 Hz, 1H), 6.16 (dd, J = 8.4, 2.6 Hz, 1H), 3.75 (t, J = 6.5 Hz, 2H), 1.57 (h, J = 7.4 Hz, 2H), 0.75 (t, J = 7.4 Hz, 3H).

*N-(4-(4-chloro-2-((4-chloro-3-(trifluoromethyl)phenyl)sulfonamido)phenoxy)-3-propoxyphenyl)acrylamide (**13**).* To a stirring solution of **12** (0.116 g, 0.22 mmol) in dichloromethane (25 mL) was added in dropwise fashion acryloyl chloride (20 µL, 0.24 mmol, 1.1 equiv. in 20 mL dichloromethane) and the reaction was stirred at room temperature for four hours. Upon completion, solvents were evaporated *in vacuo* and the crude product was purified by column chromatography eluting with 0-1% MeOH in DCM. Yield: 16% (20 mg, 0.03 mmol). ^{1}H NMR: (400 MHz, $CDCl_3$) δ 8.11 (d, J = 2.2 Hz, 1H), 7.85 (dd, J = 8.4, 2.3 Hz, 1H), 7.71 – 7.62 (m, 2H), 7.56 (d, J = 8.4 Hz, 1H), 7.48 (s, 1H), 7.38 (s, 1H), 6.96 (dd, J = 8.8, 2.5 Hz, 1H), 6.77 (dd, J = 8.6, 2.4 Hz, 1H), 6.67 (d, J = 8.6 Hz, 1H), 6.56 (d, J = 8.8 Hz, 1H), 6.46 (dd, J = 16.8, 1.2 Hz, 1H), 6.25 (dd, J = 16.8, 10.2 Hz, 1H), 5.81 (dd, J = 10.2, 1.2 Hz, 1H), 3.85 (t, J = 6.6 Hz, 2H), 1.60 (h, J = 7.2 Hz, 2H), 0.78 (t, J = 7.4 Hz, 3H). LC–MS (ESI) *m/z* calcd for $C_{25}H_{21}Cl_2F_3N_2O_5S$ $[M + H]^+$ 589.06, found 589.10, 591,10. HPLC: 12.5 min, purity 96%.

Biology

Chemicals and reagents. Compound **1** was synthesized in-house according to Ortiz-Zacarías *et al.* (2021).[1] CCR2-RA-[*R*] was purchased from MedChemExpres (Monmouth Junction, NJ). Bovine serum albumin (BSA, fraction V) and iodoacetamide were from purchased Sigma Aldrich (St. Louis, MO). [^{3}H]-CCR2-RA-[*R*] (specific activity 59.6 Ci mmol^{-1}) was custom-labelled by Vitrax (Placentia, CA). AF647-N_3 was obtained from Jena Bioscience (Jena, Germany) and Pierce™ Avidin agarose beads (cat #11846734) was ordered from Thermo Scientific (Waltham, MA). Chymotrypsin and PNGase F were ordered from Promega (Madison, WI). O-glycosidase (P0733S) and α2-3,6,8 Neuraminidase (P0720S) were purchased from New England Biolabs (MA). All other chemicals and reagents were purchased from commercial sources.

Cell culture and membrane preparation. HEK293T cells (ATCC, Manassas, VA) were cultured in DMEM – high glucose supplemented with 10% (v/v) fetal calf serum, 200 IU/mL penicillin, 200 µg/mL streptomycin and 2 mM glutamine. Chinese hamster ovary (CHO) cells were kindly provided by Hans den Dulk (Leiden University, the Netherlands, originally obtained from ATCC) and cultured in DMEM/F12 supplemented with 10% (v/v) newborn calf serum, 200 IU/mL penicillin, 200 µg/mL streptomycin and 2 mM glutamine. U2OS cells (ATCC) were cultured in DMEM – high glucose supplemented with 10% (v/v) newborn calf serum, 200 IU/mL penicillin, 200 µg/mL streptomycin and 2 mM glutamine. U2OS cells stably expressing CCR2 (Invitrogen, MA) were cultured in McCoy's 5A supplemented with 10% (v/v) (dialyzed) fetal calf serum, 2 mM glutamine, 0.1 mM nonessential amino acids, 25 mM HEPES, 1 mM sodium pyruvate, 200 IU/mL penicillin, 200 µg/mL streptomycin, 100 µg/mL G418, 40−50 µg/mL hygromycin, and 125 µg/mL zeocin. HCC70 cells (ATCC) were cultured in RPMI 1640 medium supplemented with 10% (v/v) fetal calf serum, 200 IU/mL penicillin, 200 µg/mL streptomycin and 2 mM glutamine. U266b1 (ATCC) cells were cultured in suspension in RPMI 1640 medium supplemented with 10% (v/v) fetal calf serum, 200 IU/mL penicillin, 200 µg/mL streptomycin, 2 mM glutamine and 2500 mg/L glucose. Cells were cultured twice weekly by trypsinization and were kept at 80% confluence in 10 or 15 cm Ø plates at 37 °C, 5% CO_2. For membrane preparation, cells were scraped into phosphate buffer saline (PBS) and centrifuged for 5 minutes at 3,000 rpm. Pellets were washed twice in 50 mM TRIS-HCl pH 7.4 for U2OS or 50 mM TRIS-HCl pH 7.4, 5 mM $MgCl_2$ for all other membrane types by resuspending in buffer, homogenizing for 15 seconds with the Ultra Turrax homogenizer before ultracentrifugation at 31,000 rpm for 20 minutes using the Optima LE-80 K ultracentrifuge (Beckman Coulter, Inc., Fullerton, CA). After washing, pellets were resuspended in ice-cold buffer, aliquoted and kept at -80 °C. Membrane protein concentration was determined using the Pierce BCA protein assay kit (Thermo Fisher Scientific, IL).

Transfection. HEK293T and CHO cells were transfected using the polyethylenimine (PEI) method as previously published.[1,2] In short, cells were cultured so that they were ~50% confluence on the day of transfection and kept at 37 °C, 5% CO_2. 5 µg purified HA-CCR2 (cDNA resource center, Bloomsburg, PA) or 10 µg FLAG-CCR2 or FLAG-CCR2-C70S/C75S/C232S[1] with 1 mg/mL polyethyleneimine (PEI, Polysciences Inc. Warrington, PA) in a mass ratio of 1:8 for HEK293T and 1:6 for CHO cells in a sterile 150 mM NaCl solution was added to each 10 or 15 cm Ø plate. After 24 hours at 37 °C, 5% CO_2, sodium butyrate with a final concentration of 5 mM for HEK293T was added. After an additional 24 hours, cells were used for ELISA assays or membrane preparation.

ELISA. Transfected HEK293T cells were diluted to 100 000 cells/well in medium supplemented with 5 mM sodium butyrate and plated onto poly-D-lysine coated, tissue culture treated, clear bottomed, 96-well plates. Medium was removed and cells were washed with PBS after 24 hours at 37 °C, 5% CO_2 and fixed with paraformaldehyde for 10 minutes before removing and washing twice with Tris Buffered Saline (TBS). Wells were blocked with buffer containing 2% (w/v) Bovine Serum Albumin (BSA) in Tris-

Buffered Saline, 0.1% Tween (TBST) for 1 hour at room temperature, while gently agitating. Cells were washed with TBST before addition of RabbitαHA (AHP1075, BioRad, Hercules, CA) in 0.1% (w/v) BSA in TBST with a dilution of 1:2500 for 1 hour at room temperature, while gently agitating. After washing 3 times with TBST, cells were incubated with HRP-conjugated GoatαRabbit antibody (111-035-003, Jackson ImmunoResearch Laboratories, Ely, UK) in 0.1% (w/v) BSA in TBST with a dilution of 1:2500 for 30 minutes at room temperature. Unbound secondary antibody was removed by washing 3 times with TBS, and 3,3',5,5'-Tetramethylbenzidine (TMB) was added for 5-15 minutes until a clear colour change to blue was visible. H_3PO_4 was added to stop the reaction and the plate was measured in the EnVision multilabel plate reader (PerkinElmer, Inc, Waltham, MA).

Generation of CCR2 knock-out U266B1 cell line. The CCR2 gene deletion in U266B1 cells was generated using the Alt-R™ CRISPR-Cas9 system (IDT). The two optimal pre-designed crRNA sequences 1: GACUUCUUCACCGCUCUCGUGUUUUAGAGCUAUGCU and 2: CCUGACAAUCGAUAGAUACCGUUUUAGAGCUAUGCU according to the IDT design tool were purchased from IDT together with Alt-R® CRISPR-Cas9 tracrRNA, and Alt-R® S.p. Cas9 Nuclease V3. First, the crRNA and tracRNA oligos were combined in equimolar concentrations to a final duplex concentration of 100 µM and heated at 95 °C for 5 min. Subsequently, the formed gRNA duplexes were cooled down to RT. Next, the duplexes (120 pmol) and the Cas9 enzyme (104 pmol) were combined in PBS with a total volume of 5 µL and incubated at RT for 20 min to form the ribonucleoprotein (RNP) complexes. Then, the RNP complexes were transfected into the U266B1 cell line using the Amaxa Nucleofector™ 2b device. One day prior to transfection the cells were cultured to a cell density of $5x10^5$ cells/mL. The next day, the cells were centrifuged at 200 rcf for 5 min and washed with PBS. Then, $2x10^6$ cells per condition were centrifuged at 100 rcf for 10 min. The cells were resuspended in 100 µL nucleofection solution kit C and combined with 5 µL RNP complex. The cells were nucleofected using program X-005 and afterwards allowed to recover for 30 min at RT. Simultaneously, a 12-well plate with 1.5 mL/well antibiotics free medium was preincubated at 37 °C under 5% CO_2. After recovery, the cells were transferred to the 12-well plate and incubated at 37 °C under 5% CO_2.

Validation of CCR2 knock-out U266B1 cell line and single cell isolation. Two days after transfection, 500 µL per condition cell suspension was centrifuged at 1000 rcf for 5 min and resuspended in 30 µL lysis buffer (0.45% (v/v) Tween 20, 0.45% (v/v) Triton X – 100, 2.5 mM $MgCl_2$, 50 mM KCl, 10 mM Tris-HCl pH 8.3) supplemented with proteinase K (100 µg/mL). The samples were incubated at 60 °C for 10 min and at 98 °C for 5 min. The genomic DNA extracts were directly used in PCR reactions of 100 µL with forward primer: 5' TGGCACACATGCTTTCAGG 3', reverse primer: 5' ACAACAATCAAACTGCTCCTCG 3' (custom order IDT), and Phusion Hot start II DNA Polymerase (ThermoFisher F-549S). The PCR products were purified by gel electrophoresis (1% agarose) and their DNA sequences were determined by Sanger sequencing. TIDE (Tracking of Indels by Decomposition) analysis (http://shinyapps.datacurators.nl/tide/) was performed with the sequences to determine the knock-out efficiency. For gRNA 1 (GACUUCUUCACCGCUCUCGUGUUUUAGAGCUAUGCU) and gRNA 2 (CCUGACAAUCGAUAGAUACCGUUUUAGAGCUAUGCU) this was 3.5% and 61.5%, respectively. Single cell cultures were obtained 7 days post transfection by dilution in culture medium and in 96 well plates. The cells were checked once a week under the microscope and after 9 weeks the cells were expanded in T25 flasks. Next, genomic DNA of the single cell cultures was extracted, PCR reactions were performed, Sanger sequencing data were obtained and TIDE analysis was performed as described before in order to find the correct CCR2 knock-out U266B1 single cell culture.

[^{3}H]CCR2-RA-[*R*] radioligand binding assays. U2OS cell membranes stably expressing CCR2, CHO_FLAG-CCR2(-70S/C75S/C232S), U266 and HC770 membranes were thawed and homogenized using the Ultra Turrax homogenizer (IKA-Werke GmbH & Co.KG, Staufen, Germany). Membranes were

then diluted to 20 μg/25 μL for U2OS cell membranes stably expressing CCR2, 7.5 μg/25 μL for CHO_FLAG-CCR2, 5 μg/25 μL for CHO_FLAG-CCR2-70S/C75S/C232S, 20 or 40 μg/25 μL for U266 and 20 or 40 μg/25 μL in assaybuffer containing 50 mM Tris-HCl (pH 7.4), 5 mM $MgCl_2$, 0.1% 3-((3-cholamidopropyl) dimethylammonio)-1-propanesulfonate (CHAPS) in a total volume of 100 μL. Non-specific binding was determined with 10 μM CCR2-RA-[*R*] and total binding in the presence of 1% DMSO. Competing ligands were diluted in assaybuffer with a final DMSO concentration of 1%. Membranes were incubated with increasing concentrations of competing ligand for 4 or 0 hours at 25 °C before co-incubation for 20 minutes at 25 °C with ~6.5 nM [^{3}H]CCR2-R-[*R*]. Radioligand concentrations were kept below 10% of the total radioactivity added to prevent radioligand depletion. Incubations were stopped by harvesting plates with ice-cold washbuffer (50 mM Tris-HCl, 5 mM MgCl2, 0.05% CHAPS) on pre-wetted GF/B plates using the PerkinElmer FilterMate harvester (PerkinElmer, Groningen, The Netherlands). Filters were subsequently dried at 55 °C for at least 30 minutes before the addition of 25 μL Microscint scintillation cocktail (PerkinElmer) and bound radioactivity was measured using the P-E 2450 Microbeta^2 counter (PerkinElmer).

[^{3}H]INCB3344 displacement assay. U266B1 and U266B1 CCR2 knockout (U266_CCR2-KO) cell membranes were thawed and homogenized using the Ultra Turrax homogenizer (IKA-Werke GmbH & Co.KG, Staufen, Germany). Membranes were then diluted to 20 μg/40 μL in assay buffer containing 50 mM Tris-HCl (pH 7.4), 5 mM MgCl2, 0.1% 3-((3-cholamidopropyl) dimethylammonio)-1-propanesulfonate (CHAPS) in a total volume of 100 μL. Non-specific binding was determined with 10 μM BMS CCR2 22. Membranes were incubated for 2 hours at 25 °C with ~6.5 nM [^{3}H]INCB3344. Radioligand concentrations were kept below 10% of the total radioactivity added to prevent radioligand depletion. Incubations were stopped by harvesting plates with ice-cold wash buffer (50 mM Tris-HCl, 5 mM MgCl2, 0.05% CHAPS) on pre-wetted GF/C plates using the PerkinElmer FilterMate harvester (PerkinElmer, Groningen, The Netherlands). Filters were subsequently dried at 55 °C for at least 30 minutes before the addition of 25 μL Microscint scintillation cocktail (PerkinElmer) and bound radioactivity was measured using the P-E 2450 Microbeta^2 counter (PerkinElmer).

SDS-PAGE. Sodium dodecyl sulphate–polyacrylamide gel electrophoresis (SDS-PAGE) experiments were performed with HEK293T membranes expressing HA-CCR2 (HEK293T_HA-CCR2). Samples were prepared under nitrous conditions to minimize oxidation of cysteines. Membranes were diluted in assaybuffer (50 mM Tris-HCl (pH 7.4), 5 mM $MgCl_2$, 0.1% CHAPS, degassed with nitrogen) to a final concentration of 1 mg/mL in 38 μL per sample. 1 μL of competitor or assaybuffer containing 1% DMSO was added to the membranes, which were incubated for 1 hour at 25 °C. If 1 μL restriction enzyme was added, samples were pre-incubated at 37 °C for one hour. 1 μL probe diluted in assaybuffer with a final concentration of 1% DMSO was then added to the membranes for an additional incubation step of 4 hours at 25 °C. Click mix containing 1 μM Alexa-Fluor 647-N_3 was prepared with 50 parts 100 mM $CuSO_4$, 30 parts 1 M sodium ascorbic acid (NaAsc), 10 parts 100 mM tris-hydroxypropyltriazolylmethylamine (THPTA) and 10 parts dye, added in order. After allowing the click reaction to occur for 1 hour at 25 °C, membranes were denatured with 4x Laemmli (BioRad) buffer containing β-mercaptoethanol for 15 minutes at RT. 10 μL sample was loaded onto a 12.5% acrylamide gel and run at 150-180 V for approximately 90 minutes until the blue dye had run of the gels. Images were taken with ChemiDoc™ MP imaging system (BioRad) with the Cy5 (695/55 filter) and Cy3 (605/50 filter) settings. Gels were stained for at least 18 hours in Coomassie staining before destaining the gel with 50% methanol. 10% acetic acid for 1 hour at RT and an additional 30-60 minutes in demineralized water before imaging with the ChemiDoc™ MP imaging system.

Immunoblotting. CCR2 expression in transiently HA-CCR2 transfected HEK293T cell membranes was determined using immunoblotting. Membranes were diluted to 1 mg/mL protein and homogenized

using the Ultra Turrax homogenizer (IKA-Werke GmbH & Co.KG, Staufen, Germany). Proteins were denatured in 4x Laemmli buffer (BioRad) buffer containing β-mercaptoethanol for 15 minutes at RT. 10 µL sample was loaded onto a 12.5% acrylamide gel and run at 150-180 V for approximately 90 minutes until the blue dye had run of the gels. Proteins were transferred unto a Hybond-Enhanced Chemiluminescence (ECL) membrane using the BioRad Trans-Blot Turbo for 7 minutes at 2.5 A. The blot was blocked with 5% BSA in TBST for 1 hour at RT. The primary antibody RabbitαCCR2 (Abcam ab227236) was added with a dilution of 1:5000 in 1% BSA in TBST over night at 4 °C. The membrane was washed three times with TBST for 20 minutes, before subsequent addition of the secondary antibody Horseradish Peroxidase- (HRP) conjugated GoatαRabbit antibody (Jackson ImmunoResearch Laboratories) in 1% BSA in TBST for 1 hour at RT. The membrane was washed twice with TBST and once with TBS, before addition of the of the ECL Western Blotting reagent (GE Healthcare, the Netherlands) for 5 min at RT while agitating in the dark. Images were taken with the ChemiDoc^{TM} MP imaging system.

Affinity-based pull down proteomics. Affinity-based pull down proteomics was performed as previously.[3] In short, 2 mg/mL HEK293T_HA-CCR2 membranes suspended in assaybuffer (see [^{3}H]CCR2-RA-[*R*] radioligand binding assays) were incubated with 1 µM probe **6c** or 1% DMSO for 4 hours at RT while shaking at 650 rpm. In the experiments of Figure S8, the samples were pre-incubated with 10 µM of compound **1** for 1 h at RT, prior to addition of probe **6c**. The membrane suspension was then incubated for 1 hour at RT with click mix consisting of 35 parts 100 mM $CuSO_4$, 21 parts 1 M NaAsc, 7 parts 100 mM THPTA and 1 parts 1 mM biotin-PEG3-Azide (Sigma Aldrich). Proteins were denatured by addition of SDS with a final concentration of 2.5% for 1 hour at RT while shaking at 650 rpm. Consecutively, MeOH, $CHCl_3$ and demineralized water were added to the suspension to precipitate the proteins according to a modified version of the previously reported $CHCl_3$/MeOH method.[4] Samples were centrifuged for 10 minutes at 1500 rcf after which the upper (aqueous) layer was removed. After addition of MeOH, the centrifugation step was repeated. 1% SDS buffer containing 25 mM NH_4HCO_3 was added to the pellet and samples were sonicated (Branson Sonifier; 3x5 s, 15% amplitude) to fully resuspend the pellet. Samples were reduced with 0.5 M dithiothreitol (DTT) for 15 minutes at 65 °C while shaking at 650 rpm. To alkylate samples 0.25 M iodoacetamide was used for 30 minutes at RT in the dark before quenching the excess iodoacetamide with 0.5 M DTT for 15 minutes at RT while shaking at 650 rpm. Homogenized slurry containing Avidin Agarose beads (ThermoFischer cat# 11846734) was washed three times by adding PBS followed by centrifugation for 2 min at 2500 rcf. Beads were resuspended in PBS and added to the samples for overnight incubation at 4 °C to coat the beads with protein. Samples were centrifuged for 2 minutes at 200 rcf and the pellet was resuspended in PBS containing 0.1% SDS. Samples were centrifuged (2 min at 2500 rcf) and washed three times with PBS and once with digestion buffer (100 mM Tris-HCl pH 8, 100 mM NaCl, 10 mM CaCl2 and 2% (v/v) acetonitrile) via centrifugation (2 min at 2500 rcf).[5] The coated beads were resuspended in 250 µL digestion buffer with 1 µg chymotrypsin (Promega cat# V1061) and incubated overnight at 37 °C while shaking at 1000 rpm to fragmentize proteins. Next, samples were quenched by the addition of 12 µL formic acid and samples were loaden unto Bio-Spin columns (Bio-Rad cat# 7326204) to remove the beads by centrifugation (2 min, 600 rcf). Peptides were desalted using the StageTips method and concentrated with an Eppendorf concentrator.[5,6] Dry peptides were stored at -20 °C until measurement.

The peptides were reconstituted in LCMS solution (H_2O:ACN:Formic Acid (FA) 97:3:0.1) containing 10 fmol/µL enolase digest (Waters cat #186002325). The first pull-down samples (Exp 1; Figure 5) were measured in the following manner:

The desalted peptides solution was separated on an UltiMate 3000 RSLCnano system set in a trap-elute configuration with a nanoEase M/Z Symmetry C18 100Å, 5µm, 180µm x 20 mm (Waters) trap column

for peptide loading/retention and nanoEase M/Z HSS C18 T3 100Å, 1.8µm, 75 µm x 250 mm (Waters) analytical column for peptide separation. The column was kept at 40°C in a column oven. Samples were injected on the trap column at a flow rate of 15 µl/min for 2 min with 99%A, 1%B eluent. The 85 min LC method, using mobile phase A (0.1% formic acid (FA) in ULC-MS grade water (Biosolve)) and mobile phase B (0.1% FA in ULC-MS grade acetonitrile (MeCN, Biosolve)) controlled by a flow sensor at 0.3µl/min with average pressure of 400-500 bar (5500-7000 psi), was programmed as gradient with linear increment to 1% B from t0 to t2 min, 5%B at t5 min, 22%B at t55, 40%B at t64, 90%B at t65 to t74 and 1%B at t75 to t85 min. The eluent was introduced by electro-spray ionization (ESI) via the nanoESI source (Thermo) using stainless steel Nano-bore emitters (40 mm, OD 1/32", ES542, Thermo Scientific). The QExactive HF was operated in positive mode with data dependent acquisition without the use of lock mass, default charge of 2+ and external calibration with LTQ Velos ESI positive ion calibration solution (88323, Pierce, Thermo) every 5 days to less than 2 ppm. The tune file for the survey scan was set to scan range of 350 – 1400 m/z, 60.000 resolution (m/z 200), 1 microscan, automatic gain control (AGC) of 1e6, max injection time of 50 ms, no sheath, aux or sweep gas, spray voltage ranging from 1.7 to 3.0 kV, capillary temp of 250°C and an S-lens value of 80. For the 10 data dependent MS/MS events the loop count was set to 10 and the general settings were resolution to 15,000, AGC target 1e5, max IT time 100 ms, isolation window of 1.6 m/z, no fixed first mass and normalized collision energy (NCE) of 28 eV. For individual peaks the data dependent settings were 5.00e4 for the minimum AGC target yielding an intensity threshold of 5.0e5 that needs to be reached prior of triggering an MS/MS event. No apex trigger was used, unassigned, +1 and charges >+8 were excluded with peptide match mode preferred, isotope exclusion on and dynamic exclusion of 20 sec.

For technical reasons, the latter experiments (Exp 2 and 3; Figure 5 and Figure S8) were measured using a different setup, in the following manner: the peptide solution was separated with a 85 min LC method using an Thermo Scientific Vanquish™ Neo system with a Double nanoViperTM PepMapTM Neo 2 µm C18 75 µm x 150 mm column. The method was controlled by a flow sensor at 0.3 µl/min with average pressure of 300-400 bar and was programmed as a gradient with linear increment from A (0.1% formic acid in H_2O) to B (0.1% formic acid in MeCN:H_2O 8:2), starting from 1% B from t0 to t2 min, to 5% B at t5 min, 28% B at t55 min, 50% B at t64 min, 100% B from t65 min to t74 min and 1% B from t75 min to t85 min. The eluent was introduced by electro-spray ionization (ESI) via the nanoESI source (Thermo Scientific) using stainless steel Nano-bore emitters (40 mm, OD 1/32", ES542, Thermo Scientific). The Orbitrap Exploris 240 was operated in positive mode with data dependent acquisition, without the use of lock mass and a default charge of 2+. The tune file for the survey scan was set to a scan range of 350-1400 m/z, 60.000 resolution, 1 microscan, automatic gain control (AGC) of 10^6, a maximum injection time of 50 ms, no sheath, aux or sweep gas, spray voltage of 1.9 kV, capillary temp of 280 °C and an RF-lens value of 80%. For the data dependent MS/MS events the loop count was set to 15 and the general settings were resolution to 15,000, AGC target 10^4 or 10^5, maximal IT time 100 ms, isolation window of 1.6 m/z, no fixed first mass and higher-energy collisional dissociation (HCD) of 28%. No apex trigger was used, unassigned, +1 and charges >+7 were excluded with peptide match mode preferred, isotope exclusion on and dynamic exclusion of 20 seconds.

Data analysis. Data are shown as representative or as mean ± SD of at least three individual experiments, unless otherwise specified. Data analyses were performed using GraphPad Prism 9 (GraphPad software, San Diego, CA). (p)IC_{50} values were determined using the non-linear regression curve fit. Using pIC_{50} values, (apparent) pK_i values were calculated with the ChengPrusoff equation. Images of SDS-PAGE and WB experiments were analysed using the ImageLab software (6.0.1, Biorad). Pull down proteomics data was analysed using MaxQuant (version 2.4.2.0).[7] A custom made FASTA file was used for peptide identification, consisting of the reviewed (Swiss-Prot) human proteome (downloaded on October 26 2021), isoform B of the human C-C chemokine receptor type 2 (uniprot

code P41597-2) and the background proteins bovine serum albumin (P02767), chicken avidin (P02701), yeast enolase (P00924), bovine chymotrypsinogen (P00766) and Streptomyces streptavidin (P22629). Changes to the standard MaxQuant settings were made, including setting Oxidation (M) and Acetyl (Protein N-term) as variable and Carbamidomethyl (C) as fixed modification. The digestion enzyme was set to Chymotrypsin+ with 3 max. missed cleavages. The peptide length was set to be between 7 and 25 with a max. peptide mass of 4600 Da. Contaminants were included. A false discovery rate (FDR) of 0.01 was used for peptide-spectrum match (PSM) FDR, protein FDR and site decoy FDR and the minimum amount of peptides for protein identification was set to 3. Label-free quantification was chosen with a LFQ min ratio count of 1 and 'Fast LFQ' enabled. 'Match between runs' was enabled with a match time window of 0.7 minutes and an alignment time window of 20 minutes. The output tables 'peptides.txt' and 'proteingroups.txt' files were used for further analysis. Perseus (version 2.0.11)[8] was used for data analysis of the proteingroups.txt file. In brief, proteins only identified by site, reverse hits and potential contaminants were removed. Missing values were replaced from a normal distribution using the standard settings of Perseus. In case of the competition experiments (Figure S8), the median LFQ values of two separate experiments were calculated prior to the abovementioned further analysis with Perseus. The remaining LFQ values were used to calculate fold shifts and significance using the volcano plot function in GraphPad Prism 9. Log_2(ratio) values show the ratio between the positive samples and control samples (probe/vehicle), positive samples and samples that were pre-incubated with **1** (probe/(probe+**1**)), and samples that were pre-incubated with **1** and control sampels ((probe+**1**)/vehicle).

NMR spectra

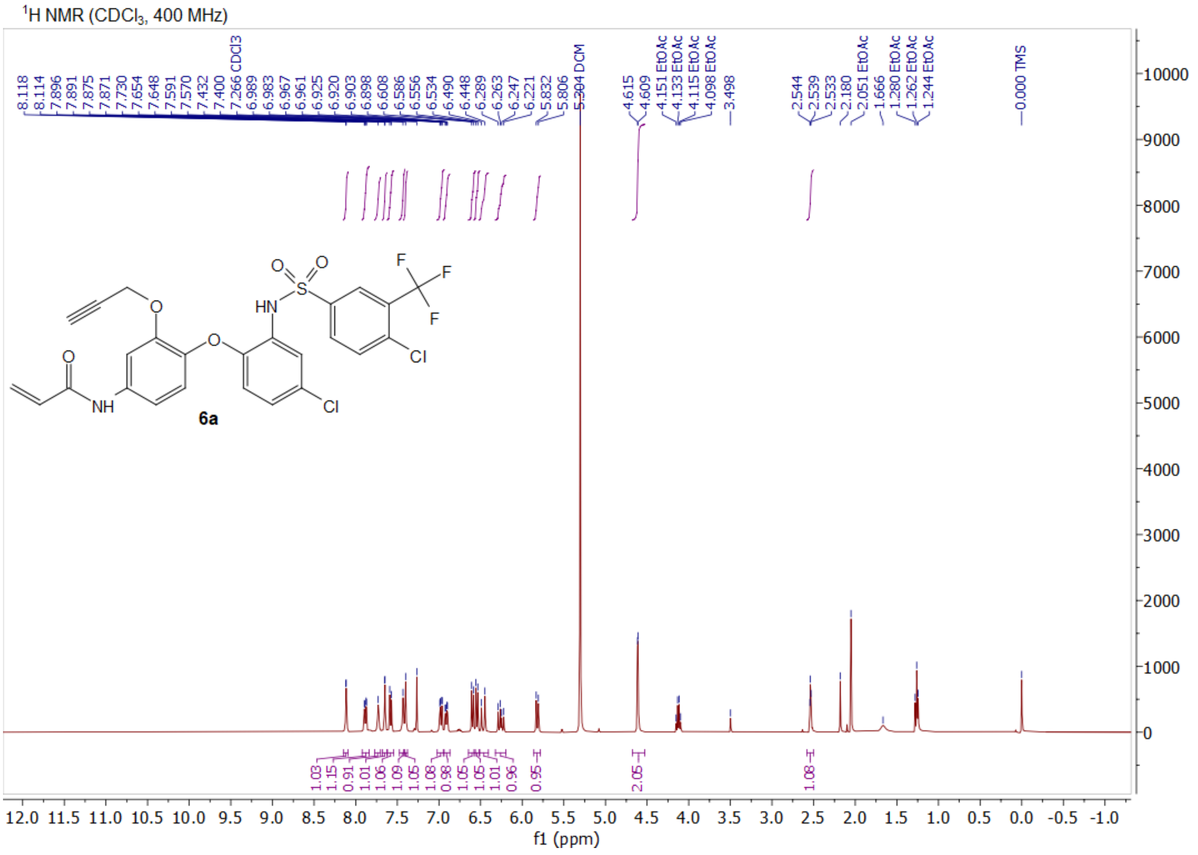

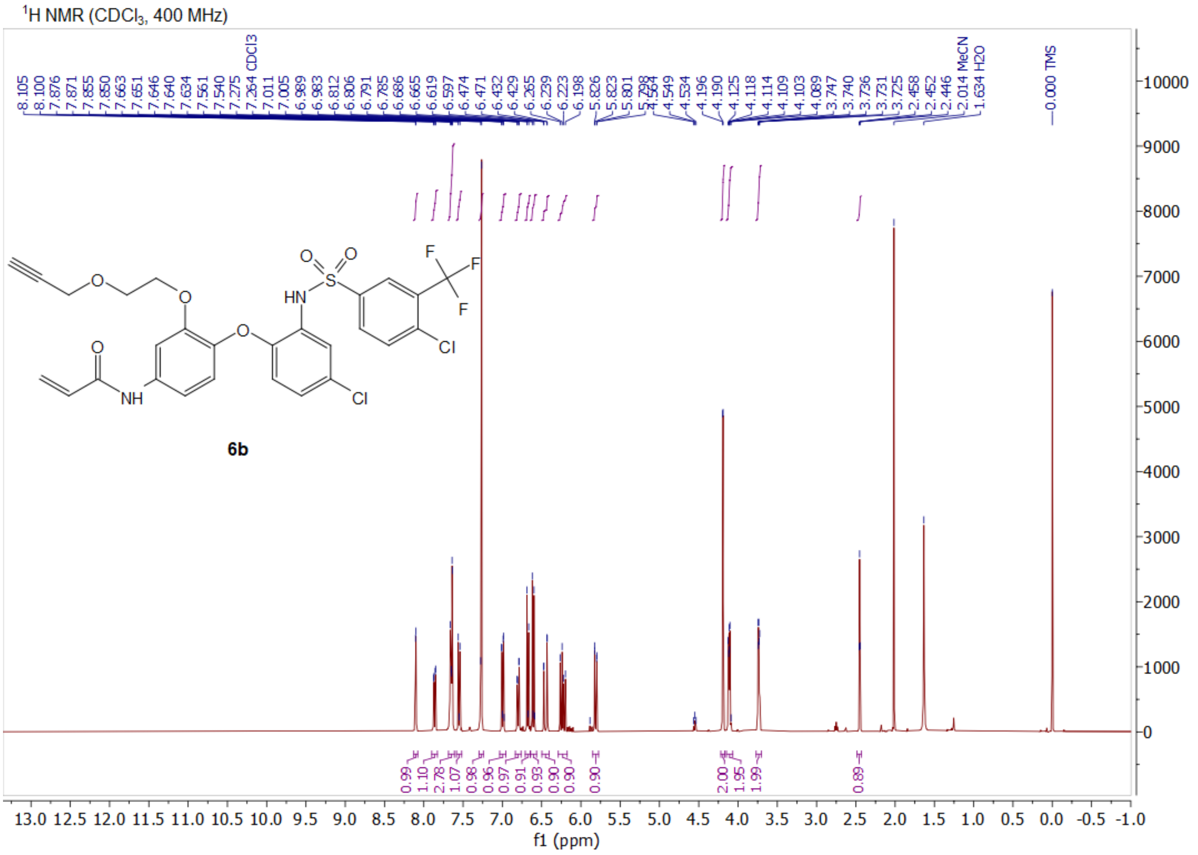

^{1}H NMR ($CDCl_3$, 400 MHz)

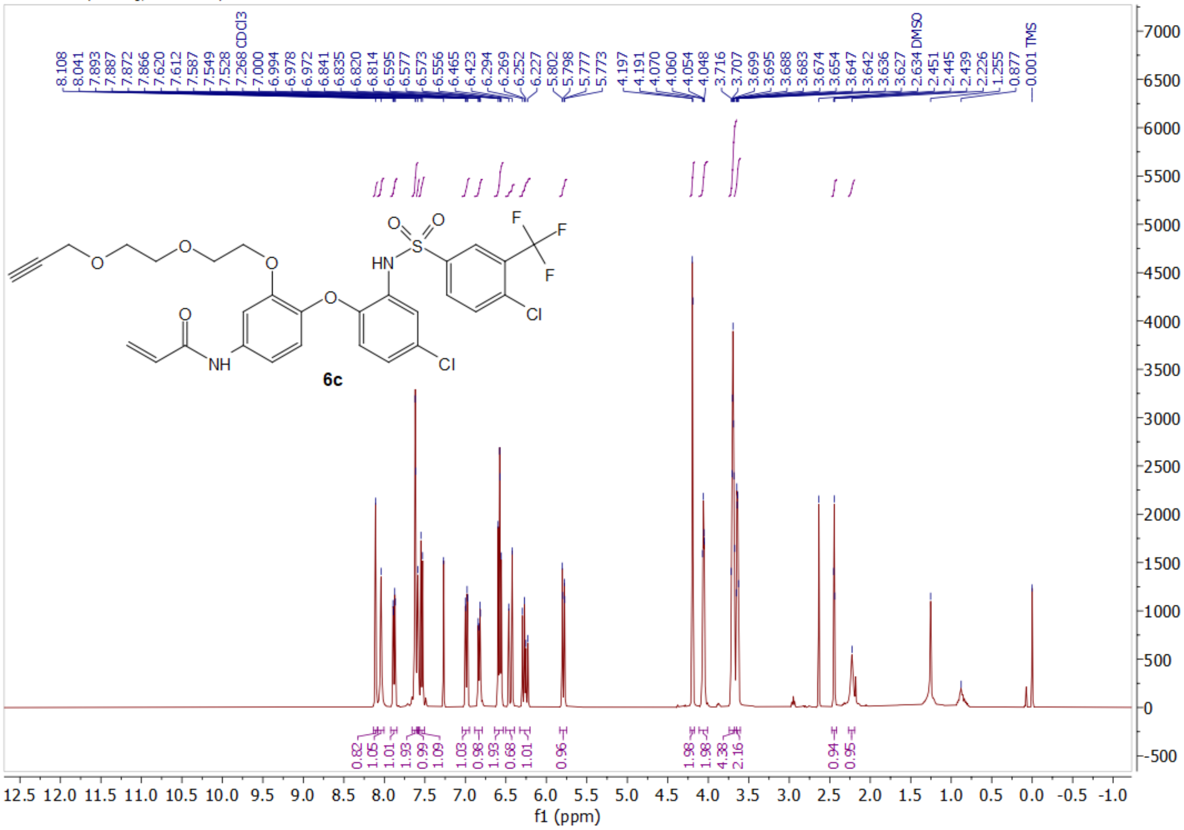

^{1}H NMR ($CDCl_3$, 400 MHz)

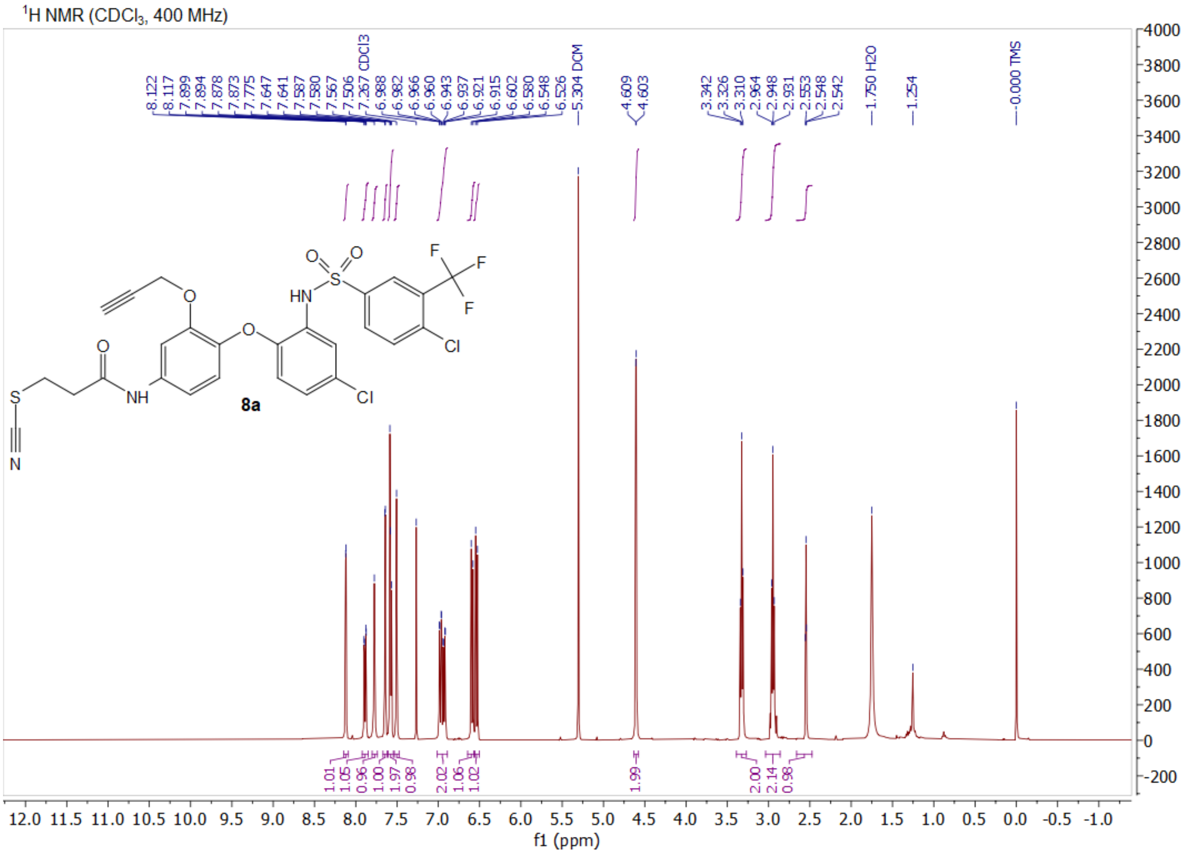

^{1}H NMR ($CDCl_3$, 400 MHz)

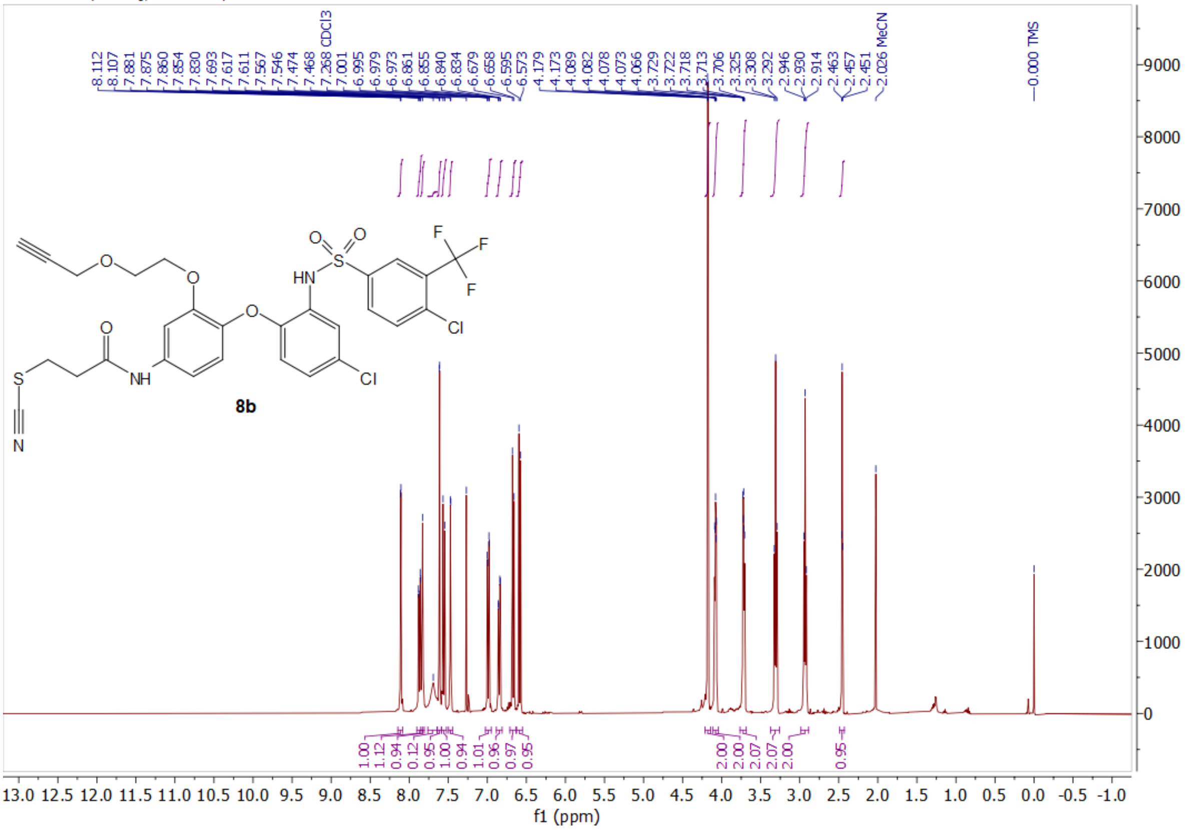

^{1}H NMR ($CDCl_3$, 400 MHz)

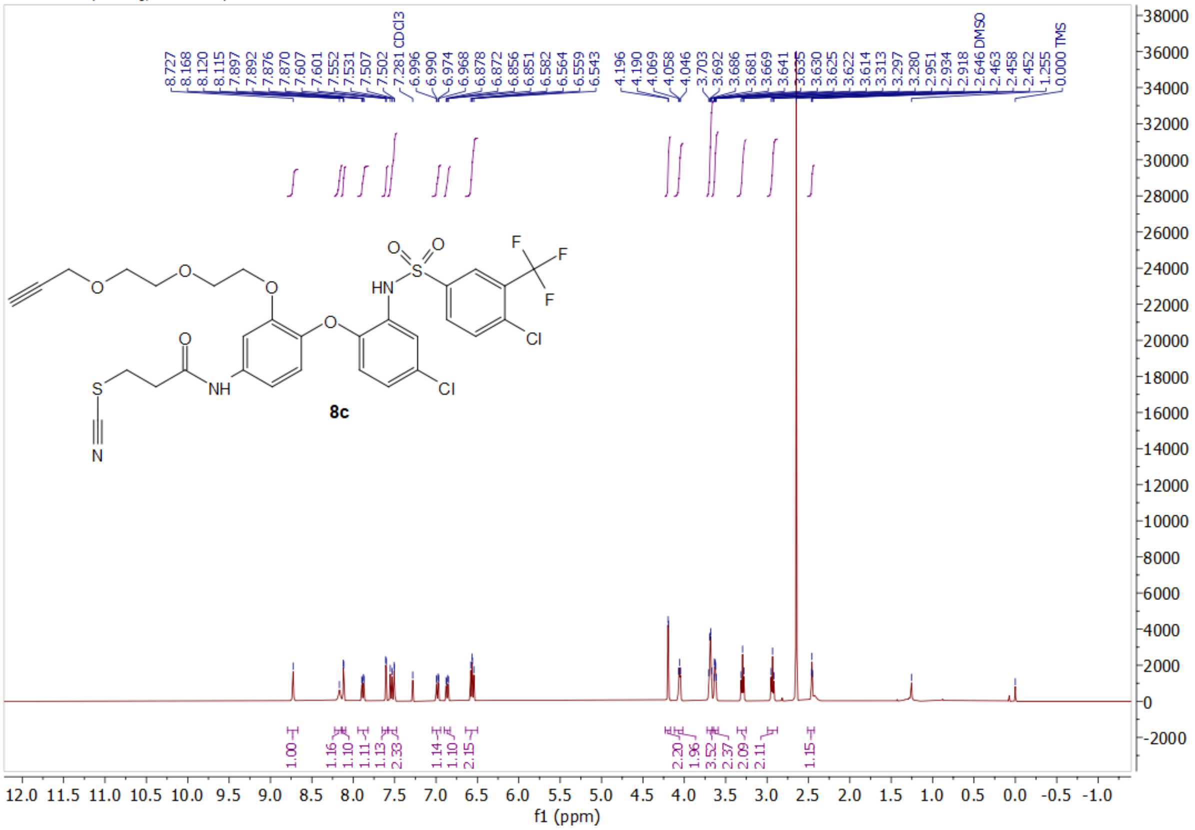

^{1}H NMR ($CDCl_3$, 400 MHz)

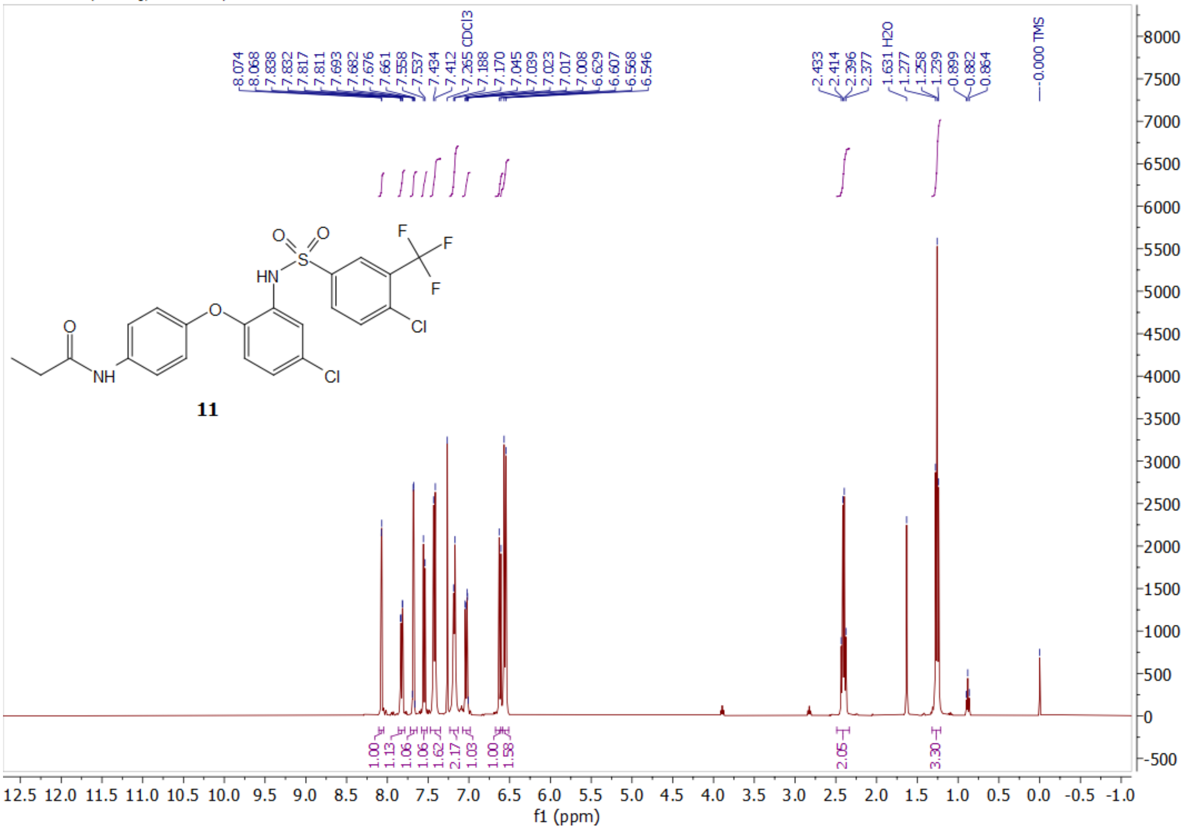

^{1}H NMR ($CDCl_3$, 400 MHz)

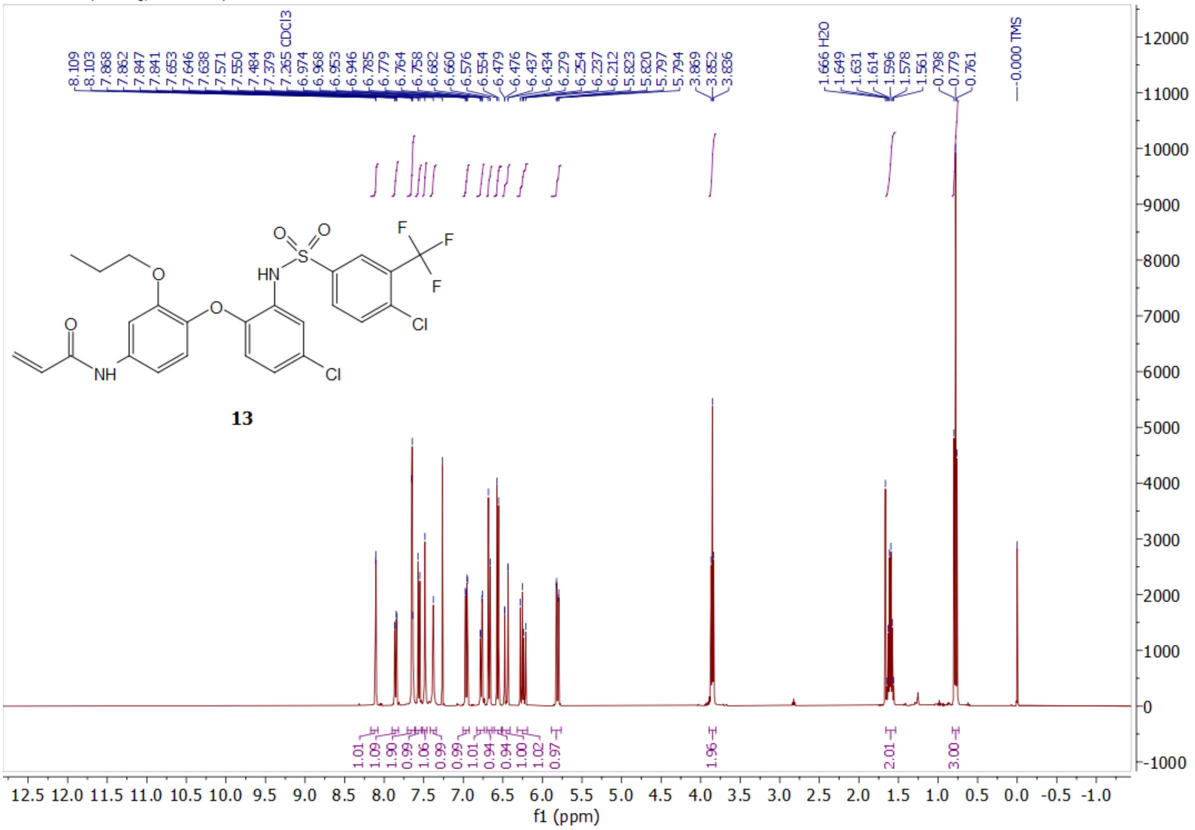

HPLC for purity analyses

Compound 6a

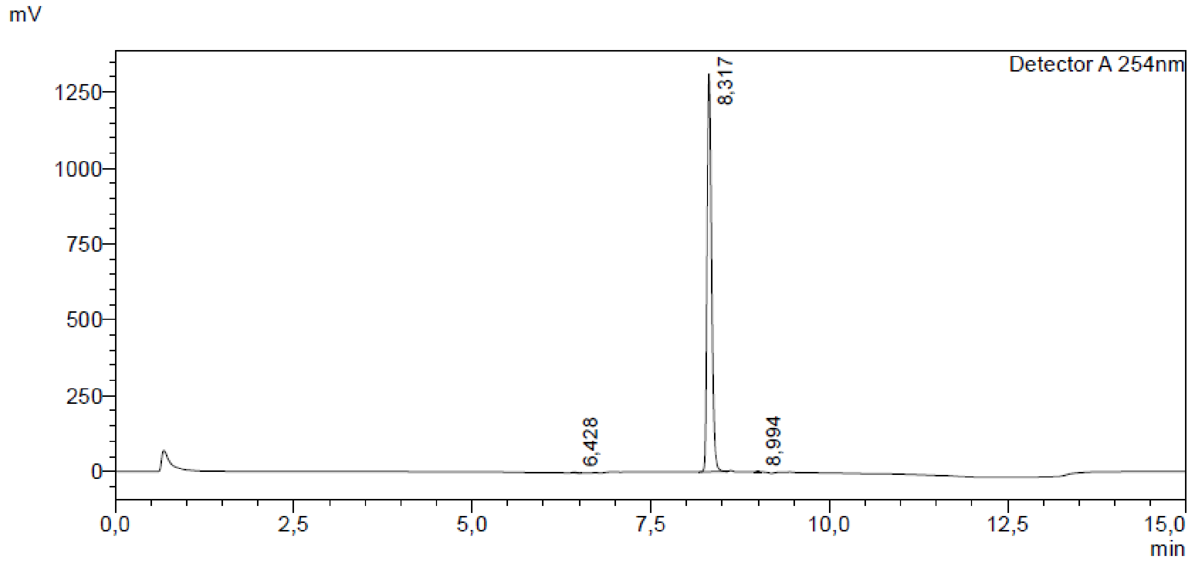

<Peak Table>

Detector A 254nm

Peak#	Ret. Time	Area	Height	Conc.	Unit	Mark	Name
1	6,428	4444	1373	0,076		M	
2	8,317	5841680	1313109	99,725		M	
3	8,994	11686	3342	0,199		M	
Total		5857810	1317824				

Compound 6b

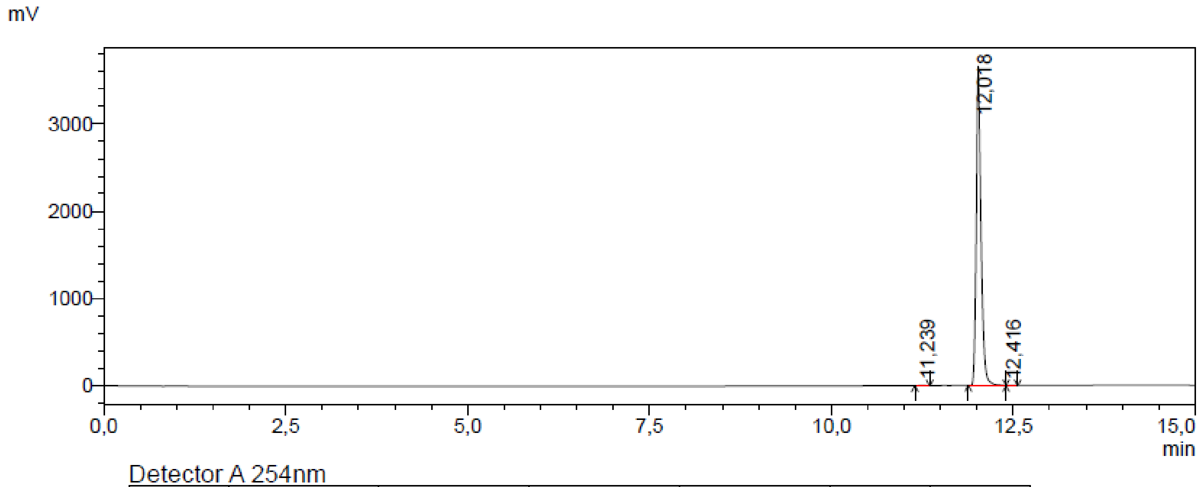

Detector A 254nm

Peak#	Ret. Time	Area	Height	Conc.	Unit	Mark
1	11,239	21755	4078	0,126		M
2	12,018	17178493	3659656	99,833		M
3	12,416	7035	1466	0,041		V M
Total		17207283	3665200	100,000		

Compound/probe 6c

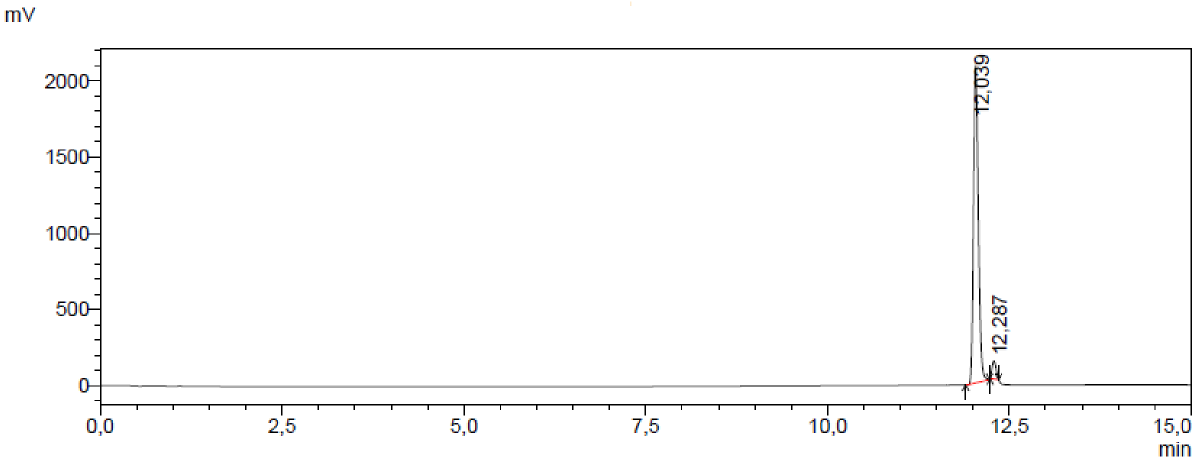

Detector A 254nm

Peak#	Ret. Time	Area	Height	Conc.	Unit	Mark
1	12,039	9886207	2075023	95,679		M
2	12,287	446440	117079	4,321		M
Total		10332646	2192102	100,000		

Compound 8a

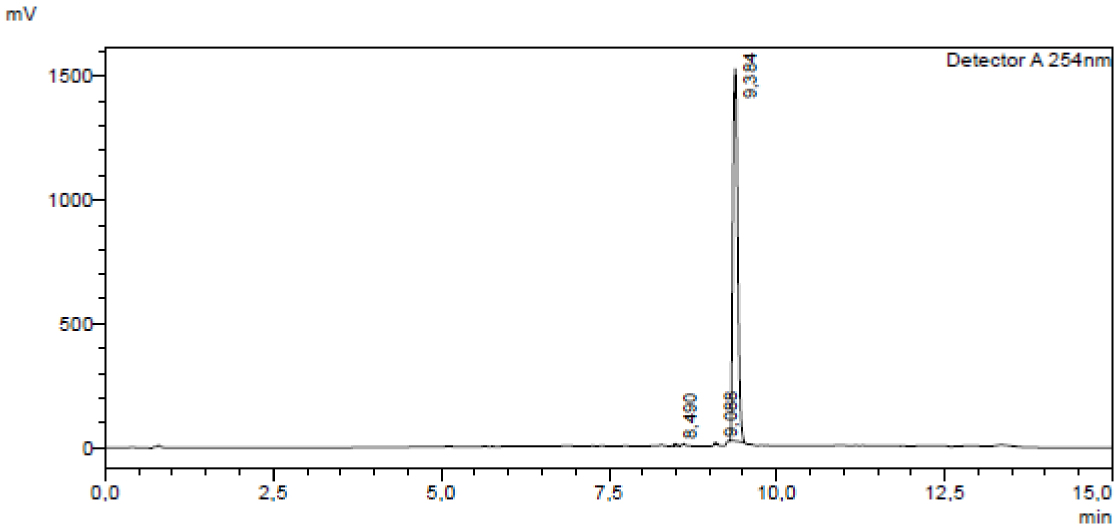

<Peak Table>

Detector A 254nm

Peak#	Ret. Time	Area	Height	Conc.	Unit	Mark	Name
1	8,490	17722	6277	0,232		M	
2	9,088	12965	6336	0,169		M	
3	9,384	7623226	1504073	99,599		M	
Total		7653913	1516685				

Compound 8b

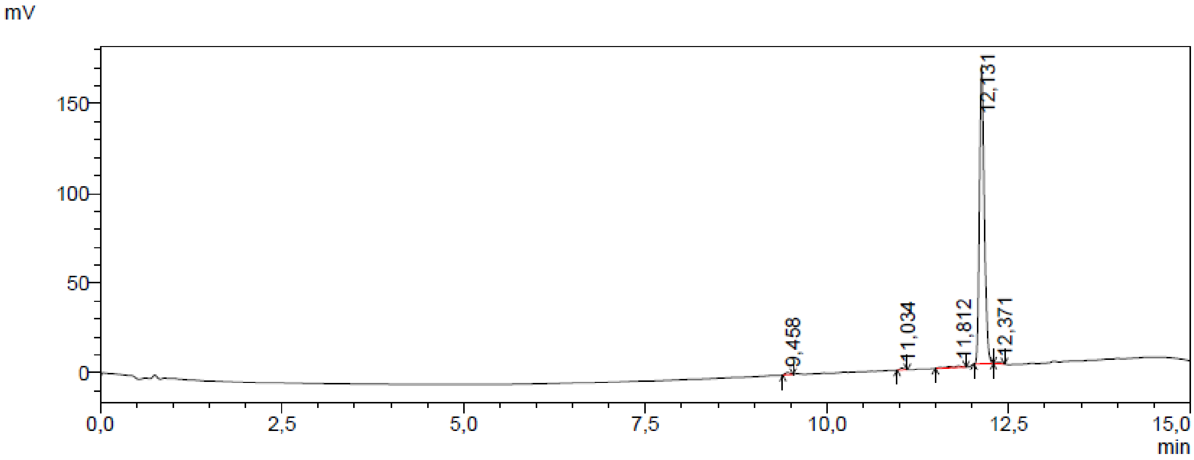

Detector A 254nm

Peak#	Ret. Time	Area	Height	Conc.	Unit	Mark
1	9,458	5427	1213	0,661		M
2	11,034	5185	1207	0,631		M
3	11,812	12129	880	1,476		M
4	12,131	795414	166739	96,817		M
5	12,371	3413	627	0,415		M
Total		821568	170666	100,000		

Compound 8c

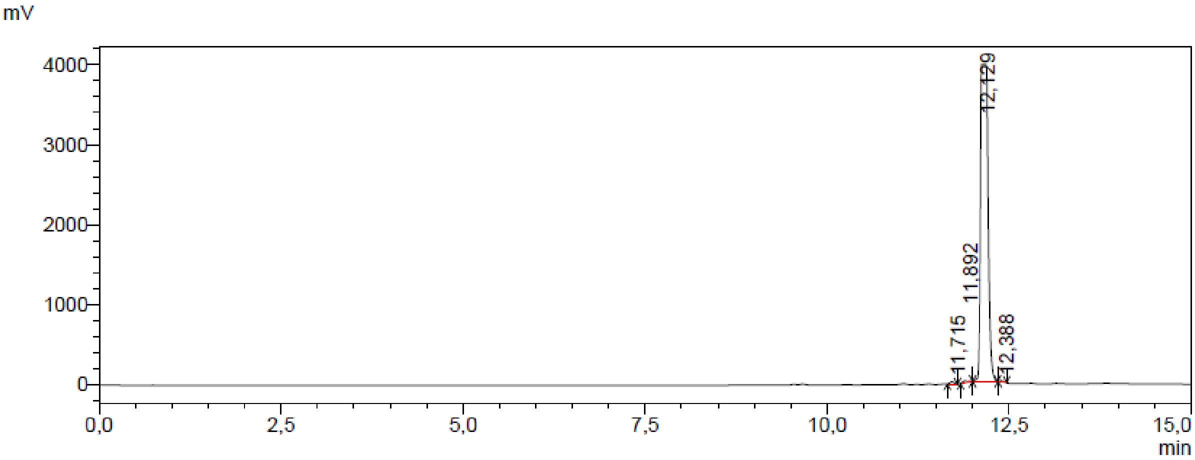

Detector A 254nm

Peak#	Ret. Time	Area	Height	Conc.	Unit	Mark
1	11,715	112459	26915	0,411		M
2	11,892	129019	25087	0,472		M
3	12,129	27056090	3953721	98,955		M
4	12,388	44370	10509	0,162		M
Total		27341938	4016231	100,000		

Compound 11

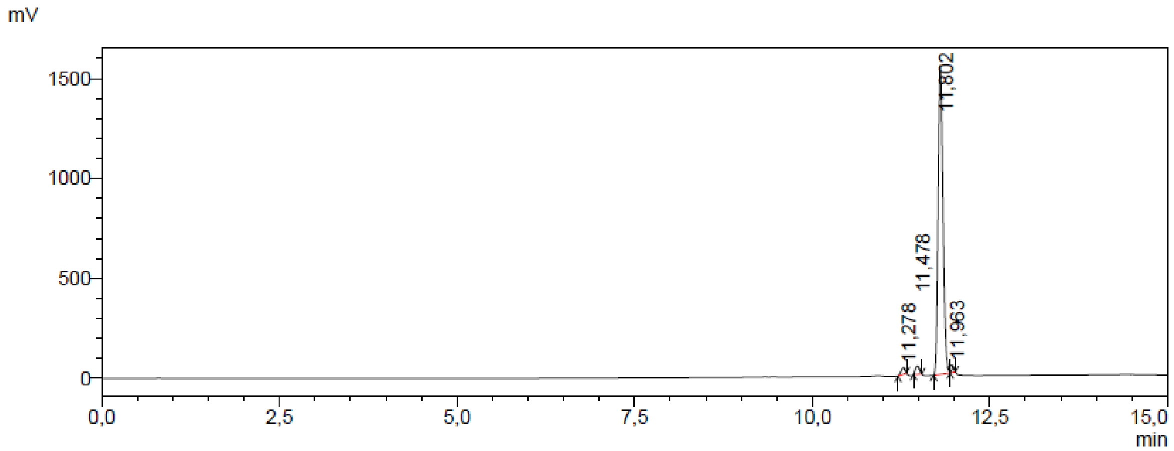

Detector A 254nm

Peak#	Ret. Time	Area	Height	Conc.	Unit	Mark
1	11,278	114085	29917	1,463		M
2	11,478	160293	41393	2,055		M
3	11,802	7417238	1543578	95,102		M
4	11,963	107621	37337	1,380		V M
Total		7799236	1652224	100,000		

Compound 13

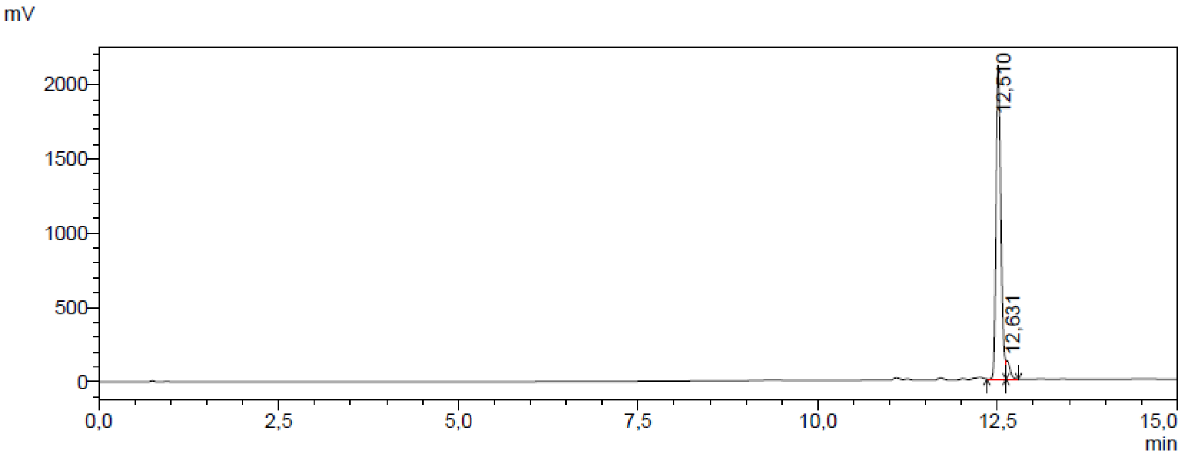

Detector A 254nm

Peak#	Ret. Time	Area	Height	Conc.	Unit	Mark
1	12,510	10089485	2115602	95,624		M
2	12,631	461762	123045	4,376		V M
Total		10551247	2238647	100,000		

References

(1) Ortiz Zacarías, N. V; Chahal, K. K.; Šimková, T.; van der Horst, C.; Zheng, Y.; Inoue, A.; Theunissen, E.; Mallee, L.; van der Es, D.; Louvel, J.; IJzerman, A. P.; Handel, T. M.; Kufareva, I.; Heitman, L. H. Design and Characterization of an Intracellular Covalent Ligand for CC Chemokine Receptor 2. *J. Med. Chem.* **2021**, *64* (5), 2608–2621.

(2) den Hollander, L. S.; Béquignon, O. J. M.; Wang, X.; van Wezel, K.; Broekhuis, J.; Gorostiola González, M.; de Visser, K. E.; IJzerman, A. P.; van Westen, G. J. P.; Heitman, L. H. Impact of Cancer-Associated Mutations in CC Chemokine Receptor 2 on Receptor Function and Antagonism. *Biochem. Pharmacol.* **2023**, *208*, 115399.

(3) Beerkens, B. L. H.; Koç, Ç.; Liu, R.; Florea, B. I.; Le Dévédec, S. E.; Heitman, L. H.; IJzerman, A. P.; van der Es, D. A Chemical Biological Approach to Study G Protein-Coupled Receptors: Labeling the Adenosine A1 Receptor Using an Electrophilic Covalent Probe. *ACS Chem. Biol.* **2022**, *17* (11), 3131–3139. https://doi.org/10.1021/acschembio.2c00589.

(4) Wessel, D.; Flügge, U. I. A Method for the Quantitative Recovery of Protein in Dilute Solution in the Presence of Detergents and Lipids. *Anal. Biochem.* **1984**, *138* (1), 141–143. https://doi.org/https://doi.org/10.1016/0003-2697(84)90782-6.

(5) Rooden, E. J. van; Florea, B. I.; Deng, H.; P, B.-M.; Esbroeck, A. C. M. van; Zhou, J.; Overkleeft, H. S.; Stelt, M. van der. Mapping in Vivo Target Interaction Profiles of Covalent Inhibitors Using Chemical Proteomics with Label-Free Quantification. *Nat. Protoc.* **2018**, *13*, 752–767.

(6) Rappsilber, J.; Mann, M.; Ishihama, Y. Protocol for Micro-Purification, Enrichment, Pre-Fractionation and Storage of Peptides for Proteomics Using StageTips. *Nat. Protoc.* **2007**, *2* (8), 1896–1906. https://doi.org/10.1038/nprot.2007.261.

(7) Tyanova, S.; Temu, T.; Cox, J. The MaxQuant Computational Platform for Mass Spectrometry-Based Shotgun Proteomics. *Nat. Protoc.* **2016**, *11* (12), 2301–2319.

(8) Tyanova, S.; Temu, T.; Sinitcyn, P.; Carlson, A.; Hein, M. Y.; Geiger, T.; Mann, M.; Cox, J. The Perseus Computational Platform for Comprehensive Analysis of (Prote)Omics Data. *Nat. Methods* **2016**, *13* (9), 731–740.
